# Systematic Discovery of Pathogen Effector Functions across Human Pathogens and Pathways

**DOI:** 10.1016/j.cell.2026.06.017

**Published:** 2026-06-30

**Authors:** Tomas Pachano, He Leng, Guillaume Dugied, Travis Tribble, Vincent Loubiere, Yeojin Lee, Felix Rauh, Victor Manon, Kevin Yuan, Jocelyn Nurtanto, Alexander Schleiffer, Veronika Young, Benjamin Weller, Eleanor A. Lyons, Matthew R. Hass, Leah C. Kottyan, Matthew T. Weirauch, Juan I. Fuxman Bass, Hayley J. Newton, Alexander W. Ensminger, Pascal Falter-Braun, Jue Chen, Daniel Schramek, Alexander Stark, Mikko Taipale

**Affiliations:** 1 Research Institute of Molecular Pathology (IMP), Vienna Biocenter (VBC), Vienna, Austria.; 2 Donnelly Centre for Cellular and Biomolecular Research, University of Toronto, Ontario, Canada.; 3 Department of Molecular Genetics, University of Toronto, Ontario, Canada; 4 Centre for Molecular and Systems Biology, Lunenfeld-Tanenbaum Research Institute, Mount Sinai Hospital, Toronto, Ontario, Canada.; 5 Howard Hughes Medical Institute, Chevy Chase, MD, USA.; 6 Weill Cornell/Rockefeller/Sloan Kettering Tri-Institutional MD-PhD Program, New York, NY, USA.; 7 Institute of Network Biology (INET), Molecular Targets and Therapeutics Center (MTTC), Helmholtz Munich, German Research Center for Environmental Health, Neuherberg, Germany.; 8 Department of Microbiology and Immunology, University of Melbourne at the Peter Doherty Institute for Infection and Immunity, Melbourne, Victoria, Australia.; 9 Center for Autoimmune Genomics and Etiology, Division of Allergy & Immunology, Cincinnati Children’s Hospital Medical Center, Cincinnati, OH, USA.; 10 Department of Pediatrics, University of Cincinnati College of Medicine, Cincinnati, OH, USA.; 11 Biology Department and Bioinformatics Program, Boston University, Boston, MA, USA.; 12 Infection Program, Monash Biomedicine Discovery Institute and Department of Microbiology, Monash University, Clayton, Victoria, Australia.; 13 Department of Biochemistry, University of Toronto, Toronto, Ontario, Canada.; 14 Microbe-Host Interactions, Faculty of Biology, Ludwig-Maximilians-Universität (LMU) München, Planegg-Martinsried, Germany.; 15 Laboratory of Membrane Biophysics and Biology, The Rockefeller University, New York, NY, USA.; 16 Medical University of Vienna, Vienna Biocenter (VBC), Vienna, Austria.; 17 Lead contact

**Keywords:** Effectors, pathogens, ORFeome, high-throughput screening, functional genomics, host-pathogen interactions, human signalling pathways

## Abstract

Pathogens deploy effector proteins to exploit host cell biology, and most effector open reading frames (ORFs) are rapidly evolving and lack functional annotation. We developed the eORFeome, a scalable functional genomics platform encompassing 3,835 effector ORFs from diverse viruses, bacteria, and parasites. High-throughput barcoded screens across NFκB, apoptosis, p53, cGAS–STING and MHC-I pathways revealed novel pathway-modulating functions for hundreds of uncharacterized eORFs, unexpected activities for known effectors, and distinct pathway-specific functions encoded by single ORFs. Illustrating the power of the approach, we identify HHV6A U14 as a p53 antagonist, HHV7 U21 as a dual-function STING antagonist and MHC-I antigen display inhibitor, and adenoviral 13.6K/i-leader protein as a *de novo* evolved TAP inhibitor that suppresses MHC-I display. These results establish a general framework for systematic effector annotation, uncover new mechanisms of host–pathogen interaction across kingdoms, and highlight pathogen effectors as a versatile toolkit for rewiring and probing human cellular pathways.

## IN BRIEF

The eORFeome, a large-scale collection of open reading frames encoding viral proteins and secreted bacterial and parasite effectors, enables functional genomics across diverse pathways and pathogens. Pooled lentiviral screens identify pathogen effectors that perturb NF-κB, p53, apoptosis, STING, and MHC-I display pathways in human cells.

## Graphical Abstract

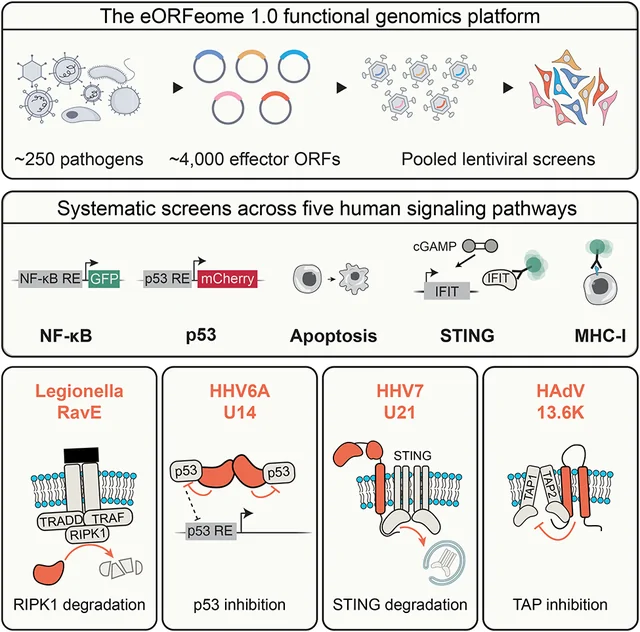

## INTRODUCTION

**Figure 1.**
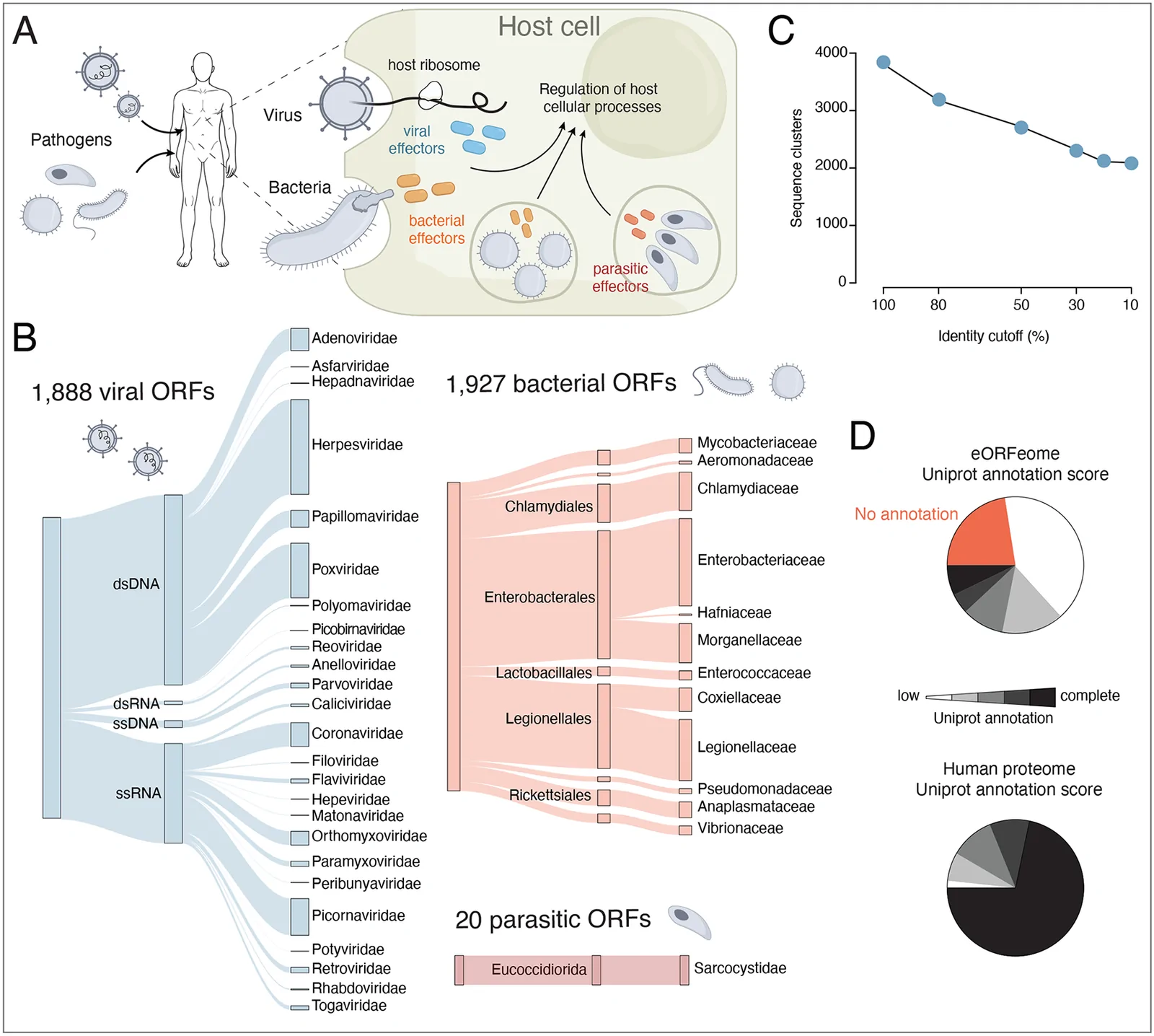
The Pan-Pathogen eORFeome Library. (A) Schematic of host cell manipulation by pathogen effectors. Diverse pathogens, including viruses, bacteria, and parasites, deploy effector proteins into host cells to hijack cellular processes, thereby promoting pathogen survival and subverting immune responses. (B) Alluvial diagrams illustrate the phylogenetic representation of the viral, bacterial and parasitic eORFs present in the eORFeome library. The flow diagrams connect taxonomic ranks - from Baltimore classification to Family for viruses, and from Order to Family for bacteria and parasites-with stream width corresponding to the abundance of ORFs from each group. (C) Sequence homology analysis of the eORFeome library. eORFs were clustered using MMseqs2^112^ at varying sequence identity cutoffs. The plot shows the total number of resulting homology clusters for each identity threshold. (D) Characterization of the eORFeome library (top) or human proteome (bottom) by UniProt annotation score. The pie chart shows the percentage of ORFs corresponding to each score. The grayscale colour key indicates the annotation level, from score 1 (white) to score of 5 (black). Orange corresponds to no entry in the UniProt database.

Pathogens express specialized gene products, known as effectors, to manipulate host molecular processes and establish conditions that are favorable for their survival and transmission.^1–3^ Many bacteria and eukaryotic parasites have evolved secretion systems that inject effector proteins into the host cell during infection, whereas viral effectors are mostly produced by the host ribosomes after viral entry (Figure 1A). By interfacing directly with host cellular machinery, effector proteins modulate immune responses, alter signaling pathways, and hijack metabolic processes to create a conducive environment for the pathogen.

At least 2,000 pathogenic viruses, bacteria, and parasites are known to infect humans^4–6^ and the increasing availability of pathogen genome sequences has facilitated the identification of millions of viral open-reading frames (ORFs)^7,8^ and the computational prediction of thousands of bacterial effectors.^9–12^ However, with the exception of a few well-studied bacteria and viruses,^13–17^ the vast majority of pathogen effectors remains uncharacterized. This leaves a large reservoir of pathogen proteins that are highly enriched for novel functions shaped by the unremitting arms race between pathogens and their hosts. Indeed, most viral proteins lack structural homologs in the AlphaFold database,^18^ pointing to unique functions and mechanisms of action. Similarly, studies of secreted bacterial effectors have revealed many unexpected molecular mechanisms, including unique post-translational modifications.^19^

The fact that thousands of sequenced pathogen ORFs have not been characterized has far-reaching scientific and societal implications. Pathogen effectors target pathways relevant to cell biology and human health, particularly to infection, inflammation, and cancer.^20–23^ Systematic functional characterization of these effectors could uncover novel host vulnerabilities and potential therapeutic entry points, pre-emptively illuminating mechanisms by which emerging pathogens manipulate human cells. Moreover, the effectors constitute a vast untapped resource of tools to perturb host cell processes, potentially via entirely novel mechanisms of action. As *evolutionarily optimized perturbagens*, they might also be more effective in modulating cellular functions than traditional perturbations such as knockouts or overexpression.

Previous studies have characterized pathogen effectors but have mostly covered a single pathogen species or relatively small collections of effectors.^24–34^ Thus, the discrepancy between the number of sequenced effector genes and those with known functions constitutes a major knowledge gap for both fundamental discovery and therapeutics. To address this gap, we present a novel approach for investigating at an unprecedented scale how diverse effectors perturb host cell functions. Rather than focusing on a single pathogen or pathway, we developed scalable assays that enable systematic functional profiling of thousands of effectors from a broad range of pathogens across a spectrum of host cellular pathways. This work introduces a powerful framework for the annotation of host perturbations elicited by pathogen effectors and the discovery of novel protein function more generally.

## RESULTS

### The effector ORFeome collection 1.0

To systematically discover the functions of diverse effectors, we assembled a large collection of effector ORFs (eORFs). Currently, this collection, which we call the effector ORFeome (eORFeome), contains 3,835 putative eORFs from viruses, bacteria, and parasites (Figure 1B, Figure S1 and Table S1). The collection includes virus-encoded ORFs from a broad phylogenetic range of viruses with representatives from all Baltimore classes (Figure 1B) and various types of ORF, including those encoding structural proteins. For polyproteins that are proteolytically processed into individual polypeptides, we included clones that correspond to final cleaved proteins. For bacteria, the eORFeome comprises ORFs from curated lists of effectors secreted into host cells by type III, type IV or type VI secretion systems, such as those from *Legionella*, *Coxiella*, and *Chlamydia*.^35–37^ We also included eORFs from the human microbiome effector ORFeome (HuMEOme_v1),^38^ which encompasses effectors from commensal gut microbes. Finally, we included 20 ORFs encoding predicted or known secreted effectors from the parasite *Toxoplasma gondii*.^39^

The eORFeome exhibits a significant sequence diversity: the 3,835 eORFs cluster into 3,208 groups at 80% sequence identity and 2,118 groups at 10% identity (Figure 1C). We recently showed that even highly similar effectors can have distinct interaction profiles and hence likely different functions^38^. Therefore, for certain pathogens (e.g. HPV, HAdV, Enteroviruses), we included highly similar homologous ORFs from multiple strains (Figure S1). Notably, only 7% of the eORFs have direct experimental evidence for protein function and structure (i.e. UniProt annotation score of 5) compared to 72% of the human proteome (Figure 1D). The eORFs also are diverse in length, ranging from 22 to 2,225 amino acids (aa), with an average size of 346 aa (Figure S2A). Collectively, the eORFeome represents a functionally underexplored resource and a unique opportunity for large-scale characterization of pathogen effectors.

### A barcoded lentiviral library and platform for inducible eORF expression and high-throughput functional screening

Inspired by screens based on human ORFeome overexpression,^22,23^ we constructed a lentiviral eORFeome library in an all-in-one vector that enables both doxycycline (dox)-inducible expression and detection of barcoded eORFs by next-generation sequencing (Figure S2B). Briefly, each eORF is followed by unique DNA barcodes that allow identification of the eORF independent of the eORF length and, moreover, increases the statistical power of screens due to an average of 200 unique barcodes per eORF (Figure S2C and S2D). Introducing this library into a starting cell population at low multiplicity of infection (MOI) ensures that most cells express only a single eORF. Controlled eORF expression by dox treatment coupled to phenotypic selection of cells by either reporter gene expression and FACS-based sorting or proliferation will enrich for cells that express eORFs with the respective function of interest. Next-generation sequencing of the barcodes allows the identification of enriched or depleted eORFs in selected cells compared to the starting population.

### A proof-of-principle screen for eORFs modulating the NF-κB pathway

**Figure 2.**
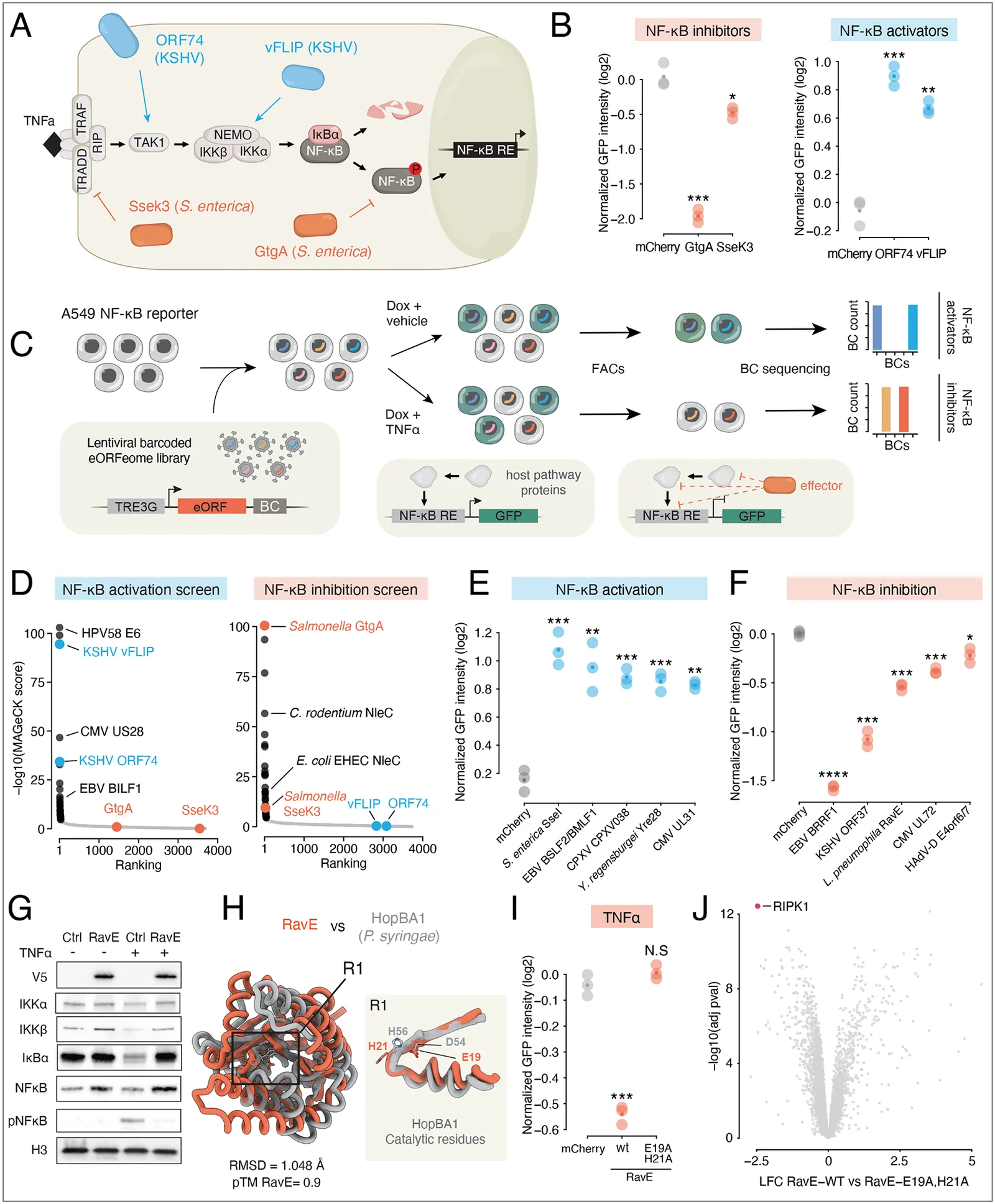
A proof-of-principle screen identifies known and novel NF-κB modulators. (A) Schematic of the NF-κB pathway. Points of intervention are shown for known inhibitory eORFs (S. enterica Ssek3 and GtgA) and activating eORFs (KSHV vFLIP and ORF74). (B) GFP intensity in an A549 NF-κB reporter cell line. Expression of the indicated ORFs was induced with dox. (Left) Cells expressing GtgA and SseK3 from Salmonella enterica, following treatment with 20 ng/mL TNFα for 16 hours to induce reporter activity. (Right) Cells expressing ORF74 and vFLIP from KSHV without TNFα treatment. The plots show the median GFP intensity, measured by flow cytometry and normalized to parallel cultures that were not treated with dox. The mCherry ORF serves as the negative control. Dots indicate the mean of replicates. (C) Schematic of the screening platform for identifying eORFs that modulate NF-κB. The A549-NF-κB reporter cell line was used to monitor pathway activity. The reporter population was transduced at a low multiplicity of infection with a dox-inducible, barcoded lentiviral eORFeome library, ensuring single eORF expression per cell. For the activation screen (top), eORF expression was induced with dox, and GFP cells (indicative of pathway activation) were enriched by FACS. For the inhibition screen (bottom), eORF expression was induced with dox, followed by stimulation with TNFα (20 ng/mL) for 16 h, and cells with reduced GFP fluorescence were enriched by FACS. For both screens, eORF-associated barcodes from sorted and input populations were recovered from gDNA and quantified by deep sequencing. MAGeCK analysis was used to aggregate enrichment scores to systematically identify candidate eORFs with significant activating or inhibitory effects on NF-κB activity. (D) Ranking plot of enrichment scores for eORFs from the NF-κB activation (left) and inhibition (right) screens. Candidate hits (black dots) are distinguished from non-hits (grey) by a threshold of FDR < 0.05 and a fold change > 1.5. Positive controls for activation (vFLIP, ORF74; blue) and inhibition (Ssek3, GtgA; orange) are indicated, and other known regulators examples are labelled. (E) Validation of candidate eORFs from the NF-κB activation screen: SseI (*S. enterica*), BSLF2+BMLF1 (EBV), CPXV038 (Cowpox virus), Yre28/WP_006817680.1(*Y. regensburgei*) and UL31 (HCMV). The assay was performed under basal conditions as in (B, right). (F) Validation of candidate ORFs from the NF-κB inhibition screen: BRRF1 (EBV), ORF37 (KSHV), RavE (*L. pneumophila*), UL72 (HCMV), and E4orf6/7 (HAdV-D). The assay was performed with TNFα stimulation as in (B, left). (G) Western blot analysis of NF-κB pathway proteins in cells expressing the novel inhibitor RavE from L. pneumophila. Cells expressing V5-tagged RavE or an mCherry control were treated with or without TNFα (20 ng/mL) for 30 min. Lysates were immunoblotted with the indicated antibodies. An anti-V5 antibody was used to confirm RavE expression, and H3 served as a loading control. (H) The structured domain of RavE (aa 1–234; orange) is shown aligned with the structure of the P. syringae effector HopBA1 (grey). A highlighted region, R1, indicates the alignment of catalytic residues from HopBA1 and RavE, and the AlphaFold 3 predicted template modeling (pTM) score for RavE is included. The Root Mean Square Deviation (RMSD) indicates the degree of structural similarity between the two proteins. (I) Comparison of the inhibitory activity of wild-type RavE and a RavE (H21A,E19A) mutant following pathway activation with TNFα. (J) Volcano plot of whole-cell mass spectrometry comparing cells expressing RavE WT versus RavE (E19A, H21A) mutant. RIPK1 is highlighted in orange. Statistical significance of panel B, E, F and I was determined by comparing each ORF to the mCherry control using multiple paired t-tests. ***padj < 0.001, **padj < 0.01, *padj < 0.05; N.S, not significant. See also Figure S3 for representative examples of the single-cell GFP fluorescence distribution.

The NF-κB pathway regulates host immunity, cell proliferation and apoptosis, making it a common target for viruses, bacteria and parasites.^40–44^ Pathogens are known to inhibit NF-κB to suppress host immune response or, conversely, activate the pathway to avoid apoptosis (Figure 2A).^40^ Thus, the NF-κB pathway provides an ideal setting for a proof-of-principle screen of the eORFeome.

We engineered an A549 reporter cell line in which an NF-κB response element drives GFP expression (see Methods). We tested the ability of the reporter to detect NF-κB inhibition by pathogen effectors by expressing two known NF-κB inhibitors from *Salmonella*. GtgA cleaves the NF-κB p65 subunit, whereas SseK3 covalently modifies the TRADD receptor with N-acetylglucosamine.^43,45^ As expected, both eORFs strongly reduced GFP signal after stimulating the NF-κB pathway with TNFα (Figure 2B and S3A). Moreover, the reporter was induced in the absence of TNFα by two known viral NF-κB activators with distinct cellular targets and mechanisms: Kaposi’s sarcoma herpesvirus (KSHV) vFLIP that activates the IκB kinase (IKK) complex, and KSHV ORF74 that activates the TAK1 kinase (Figure 2B and S3A).^46–48^ These results validate the suitability of the reporter for NF-κB modulator screens.

To identify both inhibitors and activators, we transduced the reporter cells with the eORFeome library and conducted two parallel screens (Figure 2C). In the inhibitor screen, we stimulated the pathway with TNFα and used FACS to isolate cells with low GFP levels. In the activator screen, we omitted TNFα and isolated cells with high GFP expression. Following isolation, we quantified the eORF-associated barcodes in each population by deep sequencing and computed enrichment scores.^49^

In total, the screens identified 29 activators and 42 inhibitors of NF-κB at a false discovery rate (FDR) of 0.05 and fold change > 1.5 (Table S2), including the positive controls (Figure 2D). The screens recovered multiple known NF-κB modulators (Figure 2D). These included viral and bacterial effectors, such as NleC proteins from enterobacteria and 3C proteases from enteroviruses, which inhibit NF-κB by directly cleaving the p65 subunit.^50–52^ We identified three ligand-independent vGPCRs that mimic host chemokine receptors to activate NF-κB: Epstein-Barr virus EBV BILF1, human cytomegalovirus (HCMV) US28 and HCMV UL138.^53–55^ Additionally, we found two glycoproteins implicated in NF-κB activation: HHV2 gD, a homolog of the NF-κB activator from HHV1,^56^ and EBV gH, which activates the pathway via interaction with TLR2.^57^ The activation screen also enriched for E6 proteins from multiple HPV types, which are known to induce NF-κB activity through an unknown mechanism.^58^

The screens also identified novel NF-κB regulators. To experimentally validate these candidates, we cloned a subset of novel hits and individually transduced them into the NF-κB reporter cell line. All five tested novel activator candidates strongly induced the NF-κB reporter in the absence of TNFα (Figure 2E). Furthermore, all five novel inhibitor candidates significantly suppressed NF-κB reporter activation in the presence of TNFα, with four showing strong inhibition and one displaying weaker but still statistically significant inhibition compared to control (Figure 2F).

We selected the novel inhibitor RavE to elucidate the mechanisms by which it inhibits NF-κB pathway activity. RavE (Lpg0195) is a Type IV Secretion System (T4SS) effector from *Legionella pneumophila* with unknown domain architecture and function. RavE expression in A549 cells prevented the degradation of IκBα, the cytoplasmic inhibitor of NF-κB, and the subsequent phosphorylation of NF-κB (Figure 2G) after TNFα stimulation.

Foldseek^59^ revealed structural similarity between RavE and TIKI superfamily proteins,^60^ in particular the *Pseudomonas syringae* effector HopBA1 that can activate host cell death in plants (Figure 2H).^61^ The TIKI superfamily comprises diverse esterases and proteases that share a putative active site.^60^ Structural alignment suggested that RavE residues E19 and H21, conserved among RavE homologs in other *Legionella* species (Figure S3B), might be catalytic residues. Indeed, mutating these residues to alanines abolished RavE’s ability to inhibit NF-κB activation by TNFα (Figure 2I and S3C,D).

To identify the host target(s) of RavE, we performed whole-cell mass spectrometry in cells expressing RavE, the catalytically inactive RavE mutant (E19A, H21A), or mCherry. RIPK1, a key kinase that mediates IKK complex activation downstream of TNFR1^62^, was among the most significantly downregulated proteins in RavE-expressing cells (Figures 2J and S3E and Table S4). We validated these results by western blotting (Figure S3F). Consistent with RavE specifically targeting the TNFα signaling axis, RavE did not inhibit NF-κB phosphorylation upon brief (30-minute) IL-1β stimulation (Figure S3G). However, we observed that RavE is able to block the NF-κB reporter after 24 hours of IL-1β treatment (Figure S3H). This is likely due to a feedback mechanism, where sustained IL-1β-driven NF-κB activation induces TNFα production and paracrine signaling, which is subsequently blocked by RavE.^63,64^

Thus, RavE represents a novel Legionella effector that potently inhibits the NF-κB pathway through downregulation of RIPK1. More broadly, these results establish the eORFeome screens as a powerful screening and discovery platform to deliver assay-specific hits and uncover previously unknown functional host-pathogen interactions.

### eORFeome screens uncover known and novel effectors for four additional pathways

We next performed four additional screens representing pathways that are broadly relevant: p53 signaling, STING signaling, apoptosis, and MHC-I antigen presentation. The p53 tumor suppressor regulates cell cycle progression, apoptosis, and DNA damage response and is a frequent target of viral oncogenes.^65^ The cGAS-STING pathway acts as the major innate immune sensor for cytoplasmic dsDNA. It is essential for detecting DNA viruses and intracellular bacteria, but it also plays an important role in tumor immunology and inflammation.^66^ Apoptosis is not only a conserved host defense mechanism against infection but also a key regulator of organismal development, tissue homeostasis and tumorigenesis.^67^ Finally, antigen presentation by MHC-I enables recognition of infected or transformed cells by cytotoxic T cells and thereby is a central target of immune evasion strategies employed by both pathogens and cancer cells.^68^

For each screen, we employed FACS with well-established reporters or markers: For p53, we used an A549 cell line with a p53 response element driving GFP expression, activated the pathway with the MDM2/p53 interaction inhibitor nutlin, and sorted GFP low cells. For STING, we treated U937 cells with the STING ligand 2’,3’-cGAMP and sorted cells that did not induce the cGAS-STING target IFIT1^69,70^ using an anti-IFIT1 antibody. We induced apoptosis in A549 cells with staurosporine and selected all surviving cells. Finally, we used a pan-anti-MHC-I antibody and sorted MHC-I-low A549 cells to identify inhibitors of MHC-I surface display.

**Figure 3.**
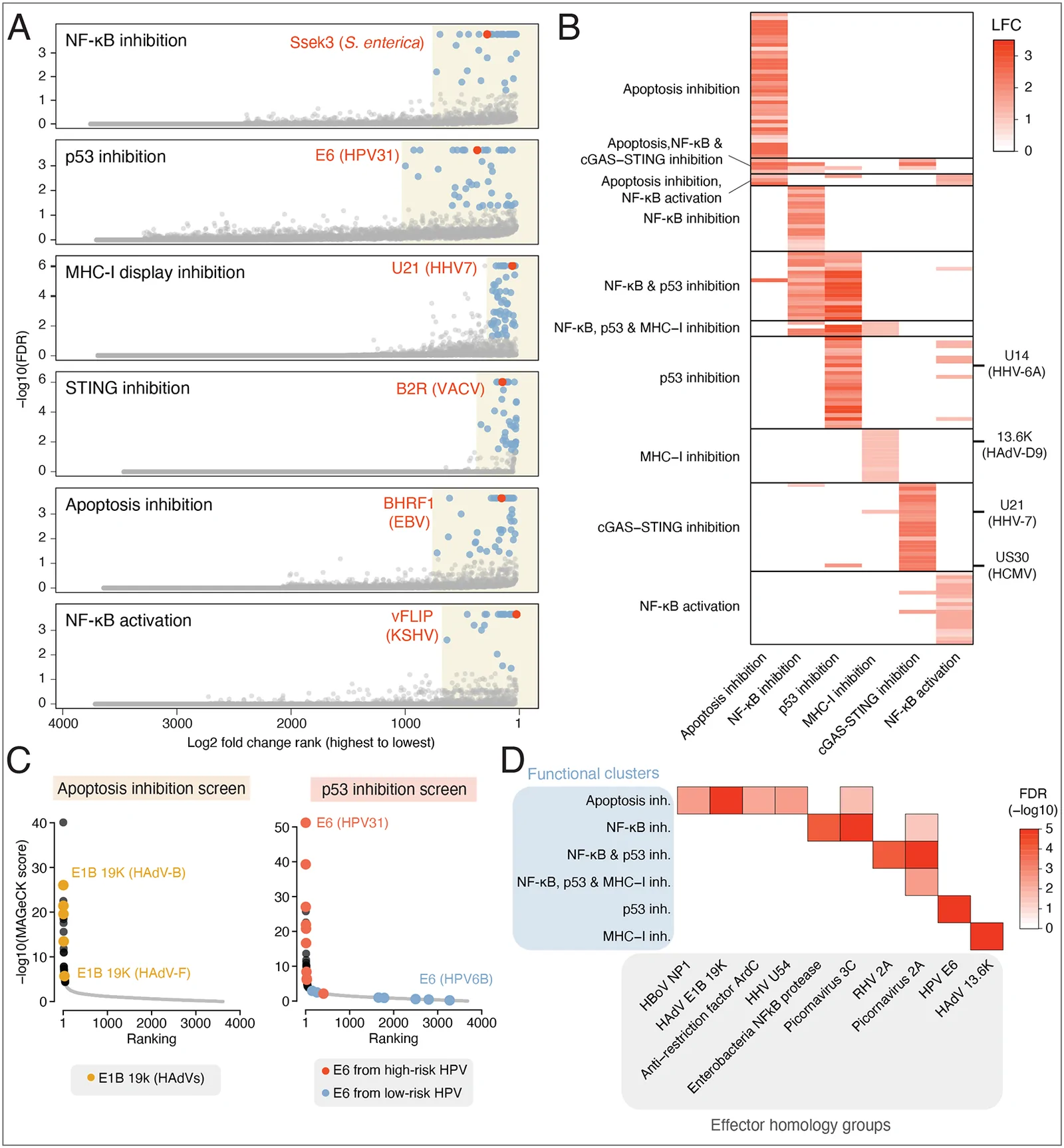
Parallel eORFeome screens uncover pathway-specific and pleiotropic effector functions. (A) Volcano plots with the results from six independent eORFeome screens. Each plot displays the enrichment (log2 fold-change; LFC) ranking on the x-axis (from highest/less enriched to lowest/more enriched) against the statistical significance (-log10 FDR) on the y-axis. Individual eORFs are represented as dots. Significant hits (FDR < 0.05 and FC > 1.5) are highlighted in blue, while a known pathway regulator is marked in orange for each screen as a positive control. (B) Self-organizing map (SOM) clustering of all significant hits (FDR < 0.05, LFC > 1) from the screens. The analysis groups eORFs into 10 distinct clusters based on their enrichment profiles (log-fold change values) across the different screens. (C) Enrichment ranking plot of eORFs from the apoptosis-inhibition screen (left) or p53-inhibition screen (right). Significant hits are marked in black. E1B 19K (HAdVs), E6 (high-risk HPVs) and E6 (low-risk HPVs) protein homologs are highlighted. (D) Enrichment of protein families (homology groups) within the functional clusters from (B). Homology groups were defined by clustering ORFs with a 30% sequence identity cutoff (MMseqs2^112^). The heatmap displays the FDR values for the enrichment of each homology group within each functional cluster. Only homology groups containing more than one ORF and with a significant enrichment (FDR < 0.05) in at least one functional cluster are shown.

In each screen, we identified several known effectors, such as HPV31 E6 inhibiting p53, Vaccinia (VACV) poxin inhibiting STING signalling, EBV BHRF1 inhibiting apoptosis, or HHV7 U21 inhibiting MHC-I display (Figure 3A, Table S2). Moreover, the screens also uncovered novel activities for hundreds of effectors, including entirely uncharacterized proteins and known effectors with unexpected additional functions.

### Parallel eORFeome screens uncover pathway-specific and pleiotropic effectors as well as shared and distinct functions for closely related eORF homologs

Our screens revealed 164 distinct hits across the six screens of which 24% (40/164) scored in multiple screens, suggesting that pathogenic effectors often target multiple processes. To discriminate pathway-specific from more pleiotropic effects, we used self-organizing map clustering to group all 164 hits into ten clusters based on their scores across the different screens (Figure 3B, Table S3). Most clusters were specific to a single pathway and contained multiple homologous proteins, suggesting conserved effector functions. For example, the apoptosis inhibition screen identified multiple Bcl2-like proteins, including five homologs of adenoviral E1B proteins known to suppress apoptosis (Figure 3C).^71^ Similarly, among the top hits of the p53 screen were several E6 proteins from papillomaviruses that are associated with a high risk of cervical cancer or head and neck cancer. In contrast, none of the E6 proteins from low-risk HPV strains were identified as screen hits (Figure 3C), consistent with the well-established oncogenic role of E6 from high-risk strains in degrading host p53.^72,73^ Thus, the screens identified both conserved and strain-specific phenotypes for effectors, highlighting the power of unbiased, sensitive screens in functional discovery.

Several protein families — defined as homology groups of eORFs sharing at least 30% sequence identity — were enriched in specific clusters (adjusted p-value < 0.05, Figure 3D). Some protein families were already linked to the respective cellular pathway, including the NF-κB-degrading zinc proteases from enterobacteria,^43,74,75^ E6 proteins from HPVs^72,73^ and E1B proteins from HAdV.^71^ However, some links were novel and could not have been predicted, including the HHV U54 tegument proteins in apoptosis or the adenoviral i-leader/13.6K proteins in MHC-I display.

Novel hits could be validated in orthogonal assays. For example, UL83 (HCMV) and BRRF1 (EBV), two hits from the apoptosis inhibition screen, suppressed PARP cleavage in etoposide-treated A549 cells relative to a GFP control (Figure S3I), confirming staurosporine-independent anti-apoptotic activity.

Thus, parallel eORFeome screens can functionally characterize hundreds of pathogen effector proteins, uncovering novel activities for individual effectors, previously unrecognized functions for entire protein families, and additional functions for known effectors. Next, we validated and characterized novel hits corresponding to each of these general lessons, selected from three different cellular pathways.

### Discovery of a novel and unexpected function: HHV6A U14 is a potent p53 antagonist

**Figure 4.**
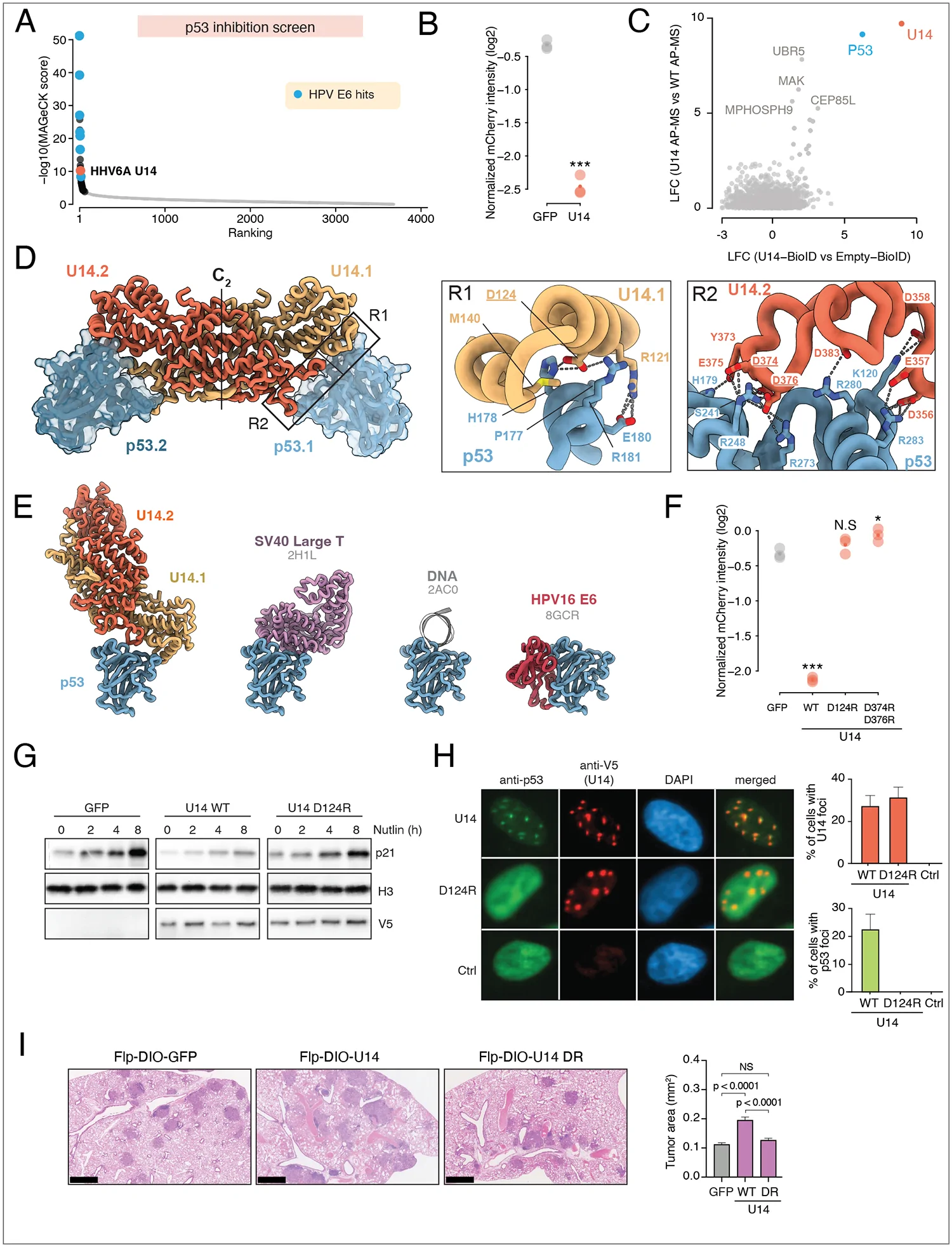
U14 from HHV6A is a potent p53 antagonist. (A) Enrichment ranking plot of eORFs from the p53-inhibition screen using a p53-mCherry reporter in A549 cells. Significant hits (FDR < 0.05 and FC > 1.5) are highlighted in black. HPV E6 proteins that scored as hits are highlighted in blue; U14 from HHV-6A is highlighted in orange. All other eORFs are shown in grey. (B) mCherry intensity in an A549 p53 reporter cell line. Expression of the novel candidate U14 (HHV-6A) was induced with dox, following treatment with nutlin (2.5 μM) for 16 hours to induce reporter activity. The plots show the median mCherry intensity, measured by flow cytometry and normalized to parallel cultures that were not treated with dox. The GFP ORF serves as the negative control. Statistical significance was determined by comparing each ORF to the GFP control using multiple paired t-tests. Dots indicate the mean of replicates. (C) Scatter plot of protein enrichment scores from BioID (x-axis) and IP-MS (y-axis) for U14 relative to an empty control. (D) AlphaFold 3 model of the U14-p53 complex. The U14 dimer is shown in orange and yellow, bound to two p53 proteins in blue and light blue. Black rectangles (R1 and R2) mark the interaction regions. The right panels show enlarged views of the interaction regions R1 and R2 from the structural model. Key amino acid residues are shown and labelled. (E) Structural models of p53 in complex with various binding partners. Shown are PDB structures of p53 bound to DNA,^133^ SV40 LTAg^77^ and HPV E6,^134^ alongside an AlphaFold 3 model of the predicted interaction between U14 dimers and p53. (F) Comparison of the inhibitory activity of wild-type U14, U14 D124R mutant or U14 D374R,D376R mutant following pathway activation with nutlin (2.5 μM) for 16 hours. (G) Western blot analysis of cells expressing GFP, V5–U14, or V5–U14 D124R, treated with nutlin for the indicated times (0–8 hours). Blots were probed for p21, V5, and H3 loading control. (H) Immunofluorescence of cells expressing an empty control, V5-tagged U14, or V5-tagged U14 D124R. Cells were stained with antibodies against p53 and V5, with DAPI for nuclear visualization. Bottom: Quantification of the percentage of cells with U14 foci (left) and p53 foci (right). Data are shown as mean ± SD from three independent experiments. (I) In vivo evaluation using a Rosa-CreERT2; FSF-KrasG12D lung adenocarcinoma mouse model. Lentivirus co-expressing U14, U14 D124R mutant, or GFP control was delivered via inhalation, followed by tamoxifen induction. Lungs were harvested 9 weeks later, H&E stained, and tumour area quantified. FACS data in all panels is shown as mean ± SD from three independent experiments. Statistical significance of panel B and F was determined by comparing each ORF to the GFP control using multiple paired t-tests. ***padj < 0.001, **padj < 0.01, *padj < 0.05; N.S, not significant. See also Figure S4 for representative examples of the single-cell mCherry fluorescence distribution.

One of the most prominent hits in the p53 inhibition screen was the U14 protein from human herpesvirus 6A (HHV6A) (Figure 4A). A prior study reported that U14 interacts with p53 but concluded that it does not inhibit p53 activity.^76^ We therefore investigated if U14 is a true p53 antagonist and determined its mechanism of action.

We expressed U14 in p53 reporter cells and observed a marked suppression of p53 reporter activation, validating U14’s role as a potent p53 antagonist (Figure 4B). Additionally, we confirmed the physical interaction between p53 and V5-tagged U14 with both affinity-purification coupled to mass spectrometry (AP-MS) and proximity-dependent biotinylation (BioID) (Figure 4C and Table S4). AlphaFold2 predicted that U14 forms a dimer with two principal regions of p53 contact (Figure 4D and S4A): Region 1 (R1) comprises residues D124 and R121 of one U14 protomer, predicted to interact with the core domain of p53 residues 177–181. Region 2 (R2) includes residues 356–376 of the second U14 protomer that are positioned to interact with the DNA-binding domain of p53 (aa 241–283). Notably, the predicted U14-p53 interaction surface is also targeted by the Large T antigen (LTAg) from SV40,^77^ a well-studied p53 antagonist unrelated to U14 (Figure 4E). Consistent with AlphaFold2 predictions, point mutations in R1 (D124R) or R2 (D374R, D376R) abolished U14’s ability to inhibit p53 reporter activity (Figure 4F and S4B–C). Like LTAg^78^, U14 did not reduce p53 protein levels (Figure S4D). Furthermore, wild-type U14 but not the D124R mutant impaired the induction of p21, a key p53 target, following nutlin treatment in A549 cells (Figure 4G), supporting the structural model and highlighting the critical role of the two regions.

To directly test whether U14 inhibits p53 DNA binding like LTAg^78^, we performed an electrophoretic mobility shift assay (EMSA). Consistent with the predicted interface, the p53 DNA-binding domain bound the p53 response element probe but not a scrambled control probe, and purified U14 inhibited probe binding in a concentration-dependent manner (Figure S4E–F). We also observed that, in ~25% of cells, U14 expression led to p53 sequestration into distinct nuclear foci, which colocalized with U14 (Figure 4H). This relocalization depended on direct U14-p53 interaction, as the U14 D124R mutant, while still forming nuclear foci, did not recruit p53 (Figure 4H). To test if p53 sequestration occurs in infected cells, we used SupT1 T-cell lymphoma cells that are susceptible to infection by a close relative of HHV6A, HHV6B that also encodes U14.^79^ While uninfected SupT1 cells showed diffuse p53 nuclear staining, SupT1 cells infected with HHV6B using a co-culture method^79^ had nuclear p53 foci in a subset of cells (Figures S4G–H), suggesting that p53 sequestration occurs also during HHV6B infection. We note that fewer cells had p53 foci during infection than after ectopic U14 expression (~3% versus ~25%), which could be partly due to inefficient HHV6B infection in vitro.^79^ We also note that in both conditions, only a fraction of cells show p53 foci while p53 is inhibited in all cells, such that the formation of foci is not required for p53 inhibition.

Next, we hypothesized that U14 might interfere with p53-mediated tumor suppression and cooperate with the Kras^G12D^ oncogene to drive tumor formation.^80^ We first validated in mouse LLC1 lung cancer cells that U14 can antagonize murine p53 in vitro (Figure S4I). To test U14’s biological impact *in vivo*, we engineered lentiviral vectors that contain the FLP recombinase and a double-floxed inverted orientation (DIO) cassette of wild-type U14, U14 D124R, or eGFP. We delivered the vectors through intranasal inhalations into the lungs of Frt-Stop-Frt (FSF)-Kras^G12D^; Rosa26-CreERT2 mice^81,82^, where a premature stop codon prevents Kras^G12D^ expression in the absence of recombination. FLP recombinase clonally activates the FSF-Kras^G12D^ allele within the lung of inhaled mice, while administration of tamoxifen allows temporal control of transgene expression via CreERT2. U14-expressing mice developed nearly double the tumor area compared to both eGFP control and the U14 D124R mutant (Figure 4I). Furthermore, mice expressing U14 showed significantly reduced overall survival compared to eGFP controls (Figure S4J). These results provide strong evidence that U14 functions as a potent p53 antagonist *in vivo*, accelerating tumorigenesis by dismantling the cell’s primary defense against oncogenic transformation. More broadly, these findings suggest a functional evolutionary convergence between dissimilar effectors of two unrelated viruses (HHV6 and SV40), both of which inhibit p53 DNA-binding activity through the same interface.

### Discovery of a new function for a known viral effector: HHV7 MHC-I inhibitor U21 is also a potent inhibitor of STING signaling

**Figure 5.**
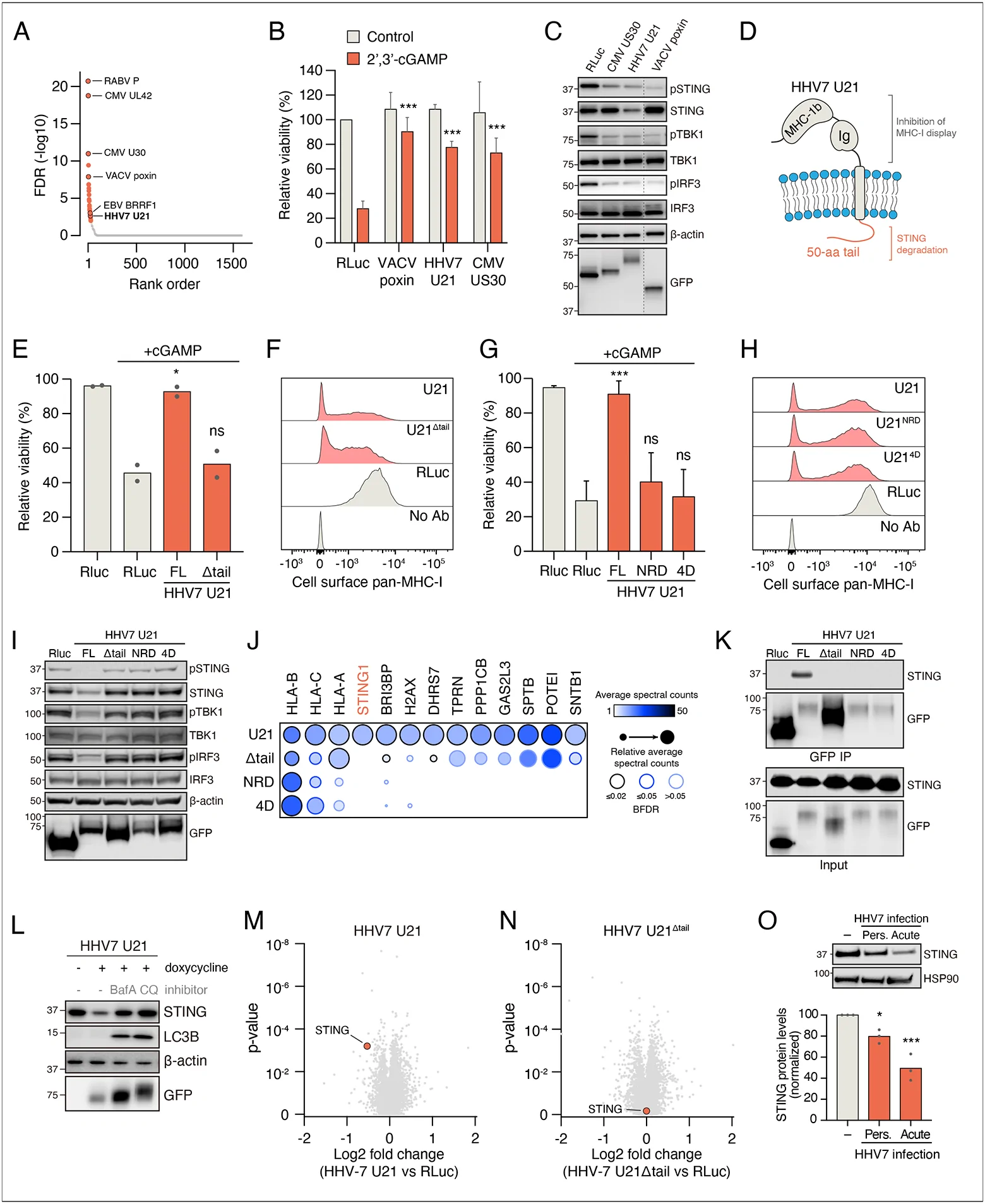
HHV7 U21 antagonizes cGAS-STING signaling by inducing STING degradation. (A) Enrichment ranking plot of eORFs from the STING inhibition screen in U937 cells. Significant hits are highlighted in red. Notable known inhibitors of STING signaling are labeled, with U21 highlighted as a novel STING inhibitor. All other eORFs are shown in grey. (B) U937 cells infected with indicated lentiviral dox-inducible GFP-tagged constructs, treated with dox and 40 μg/mL 2’,3’-cGAMP or vehicle and assessed for viability with CellTiter-Glo. (One-way ANOVA with Dunnett’s comparisons test ***padj < 0.001, n = 6 for RLuc and Poxin and n = 3 for HHV7 U21 and HCMV US30). (C) Indicated GFP-tagged proteins were expressed in cGAMP-treated U937 cells followed by western blotting for STING pathway components. (D) Schematic of HHV7 U21 protein. (E) U937 cells expressing full-length U21 (FL), U21 lacking the C-terminal tail (U21^Δtail^, aa 1–380), or Renilla luciferase (RLuc) were tested for suppression of cGAMP-induced cell death as in (B). (One-way ANOVA with Dunnett’s comparisons test *padj < 0.05, ns: not significant). (F) U937 cells expressing U21, U21^Δtail^, or RLuc were stained for cell surface MHC-I and analyzed by flow cytometry. (G) U21 and two U21 C-terminal tail mutants U21^NRD^ and U21^4D^ were assayed for their ability to suppress cGAMP toxicity as in (B). (One-way ANOVA with Dunnett’s comparisons test ***padj < 0.001, ns: not significant, n = 6). (H) U21, U21^NRD^, and U21^4D^ were assayed for MHC-I cell surface display as in (F). (I) U21 and indicated mutants were assayed for inhibition of STING pathway after cGAMP stimulation by western blotting as in (C). (J) The proximity interactomes of U21 and indicated mutants were characterized by BioID. The heat map shows proximity interactors of U21 that passed the BFDR threshold of <0.05 and spectral counts >8. The full dataset is available in Table S5 (K) Indicated GFP-tagged proteins were immunoprecipitated from U937 cells with a GFP antibody followed by western blotting with STING and GFP antibodies. (L) U21 was expressed in U937 cells that were treated with bafilomycin A (BafA), chloroquine (CQ) or left untreated and STING protein levels were analyzed by western blotting. LC3B levels were assayed to validate the activity of the compounds. (M-N) Full-length HHV7 U21 (M) or U21Δtail (N) were expressed in U937 cells, which were subjected to whole cell proteomics. (O) STING protein levels were analyzed by western blotting in uninfected SupT1 cells, SupT1 cells persistently infected with HHV7, and freshly infected SupT1 cells. (One-way ANOVA with Dunnett’s comparisons test ***padj < 0.001, *padj < 0.05).

The screen for STING pathway inhibitors identified multiple known pathway modulators, including VACV poxin, a nuclease that degrades cGAMP (Figure 5A),^83^ HCMV UL42, which inhibits STING translocation,^84^ and the Rabies virus P phosphoprotein that inhibits TBK1 and IRF3 function downstream of STING.^85,86^ Novel hits without previous connection to cGAS-STING signaling included the herpesvirus proteins HCMV US30 and HHV7 U21.

Activation of STING by cGAMP in U937 cells leads to cell death.^87,88^ We therefore assessed the ability of dox-inducible, GFP-tagged VACV poxin, HCMV US30, and HHV7 U21 to suppress cGAMP-induced cell death. Consistent with the screen, all proteins potently suppressed cGAMP-induced apoptotic cell death when expressed individually (Figure 5B and S5A). However, in contrast to poxin, which could suppress cell death induced by cGAMP but not by the non-cyclic dinucleotide STING agonist diABZI,^89^ the other two viral effectors counteracted the effect of both ligands (Figure S5B). Thus, the novel effectors function through a different mechanism than poxin.

All three effectors also inhibited cGAMP-induced phosphorylation of STING and downstream signaling components TBK1 and IRF3 (Figure 5C). Interestingly, however, US30 and U21 had different effects: US30 inhibited STING phosphorylation without affecting its levels, whereas U21 expression led to lower STING protein levels. U21 is a single-pass transmembrane domain protein with an MHC-Ib-like and Ig-like lumenal domains and a 50 amino acid unstructured cytoplasmic tail (Figure 5D).^90^ U21 was originally identified as an immunoevasin that routes MHC-I molecules to lysosomes to evade recognition by cytotoxic T cells.^91,92^ The lumenal domain is required for MHC-I redirection to lysosomes whereas the cytoplasmic tail is dispensable^93^. However, we observed that deleting the tail abolished the ability of U21 to suppress cGAMP-induced cell death (Figure 5E and S5C) while retaining MHC-I display inhibition (Figure 5F), indicating that these activities are genetically separable. There was no difference in STING mRNA levels, ruling out a transcriptional downregulation (Figure S5D). Moreover, U21 could downregulate STING levels independent of cGAMP treatment, indicating that it modulates basal STING levels (Figure S5E).

To identify functionally important residues in the U21 C-terminal tail, we compared U21 sequences from HHV7, HHV6A, and HHV6B as well as macaque herpesvirus 7 (MneHV7).^94^ We identified two conserved, mostly hydrophobic motifs adjacent to the transmembrane domain (Figure S5F). Mutating five residues in the first motif (U21^NRD^) or four hydrophobic residues to aspartic acids in the second motif (U21^4D^) phenocopied the U21 tail deletion, indicating that both motifs are required for suppressing cGAMP toxicity but dispensable in MHC-I display inhibition (Figures 5G–H). Consistent with this, all U21 tail mutants lost their ability to suppress STING phosphorylation as well as downstream phosphorylation of TBK1 and IRF3 (Figure 5I).

To test if U21 physically interacts with STING, we expressed GFP-tagged U21, U21^Δtail^, U21^NRD^, U21^4D^, and Nanoluc control in U937 cells and characterized their interactomes with AP-MS after gentle cell lysis with digitonin to preserve membrane protein interactions. Consistent with the functional assays, all constructs interacted with MHC class I molecules HLA-A, HLA-B and HLA-C (Figure 5J). In contrast, although full-length U21 robustly interacted with STING, none of the mutants did (Figure 5J). We further validated these results by co-immunoprecipitation of endogenous STING with GFP-tagged constructs. As in AP-MS, only the wild-type U21 interacted with STING (Figure 5K).

Because U21 reroutes MHC-I molecules to the lysosome, we hypothesized that it might regulate STING levels also through a lysosome-dependent pathway. We therefore treated U21-GFP expressing U937 cells with the vacuolar ATPase inhibitor bafilomycin A1 or with chloroquine that inhibits autophagosome fusion with lysosomes. Both bafilomycin A1 and chloroquine restored STING levels to that of control in HHV7 U21 expressing cells (Figure 5L), indicating that U21 induces STING degradation through a lysosome-dependent pathway.

To test if U21 more broadly induces cellular protein degradation, we conducted a whole-cell proteomics experiment in U937 cells expressing full-length GFP-tagged U21, U21^Δtail^, or Renilla luciferase as a control. After inducing the expression for 24 hours with dox, HHV7 U21 significantly upregulated only 5 proteins and downregulated 5 proteins including STING (STING, SRPK1, Hsp70, ARMH3, IFIT1) (Figure 5M and Table S5). In contrast, U21^Δtail^ expression did not affect STING levels (Figure 5N). Notably, while western blotting with antibodies against the endogenous proteins validated STING downregulation by U21 (Figure 5L and S5E), the downregulation of SRPK1, Hsp70, and ARMH3 was not validated (Figure S5G), suggesting that these are not robust targets of U21 (the interferon-inducible protein IFIT1 could not be detected in basal conditions).

Finally, to investigate if STING is downregulated during HHV7 infection, we used an in vitro co-culture infection method in which HHV7-positive SupT1 cells were co-cultured with fresh, uninfected SupT1 cells at a 1:10 ratio until cytopathic effects began to appear.^95^ Lytic infection induced robust STING downregulation of about 50%, whereas cells with persistent HHV7 infection had approximately 20% lower STING protein levels than uninfected SupT1 cells (Figure 5O). The decrease in STING protein during lytic infection could not be explained by lower transcript levels (Figures S5H–I). Thus, HHV7 can downregulate STING protein during lytic infection.

Taken together, U21 has a limited but very specific effect on the global proteome, and it uniquely downregulates STING protein via its C-terminal tail and the lysosome. It therefore represents a dual-function effector that can inhibit both adaptive and innate immune responses by targeting MHC-I molecules and STING with topologically distinct domains.

### Functional annotation of a recently evolved non-canonical eORF: Adenoviral i-leader/13.6K proteins inhibit surface MHC-I expression via TAP transporter inhibition

**Figure 6.**
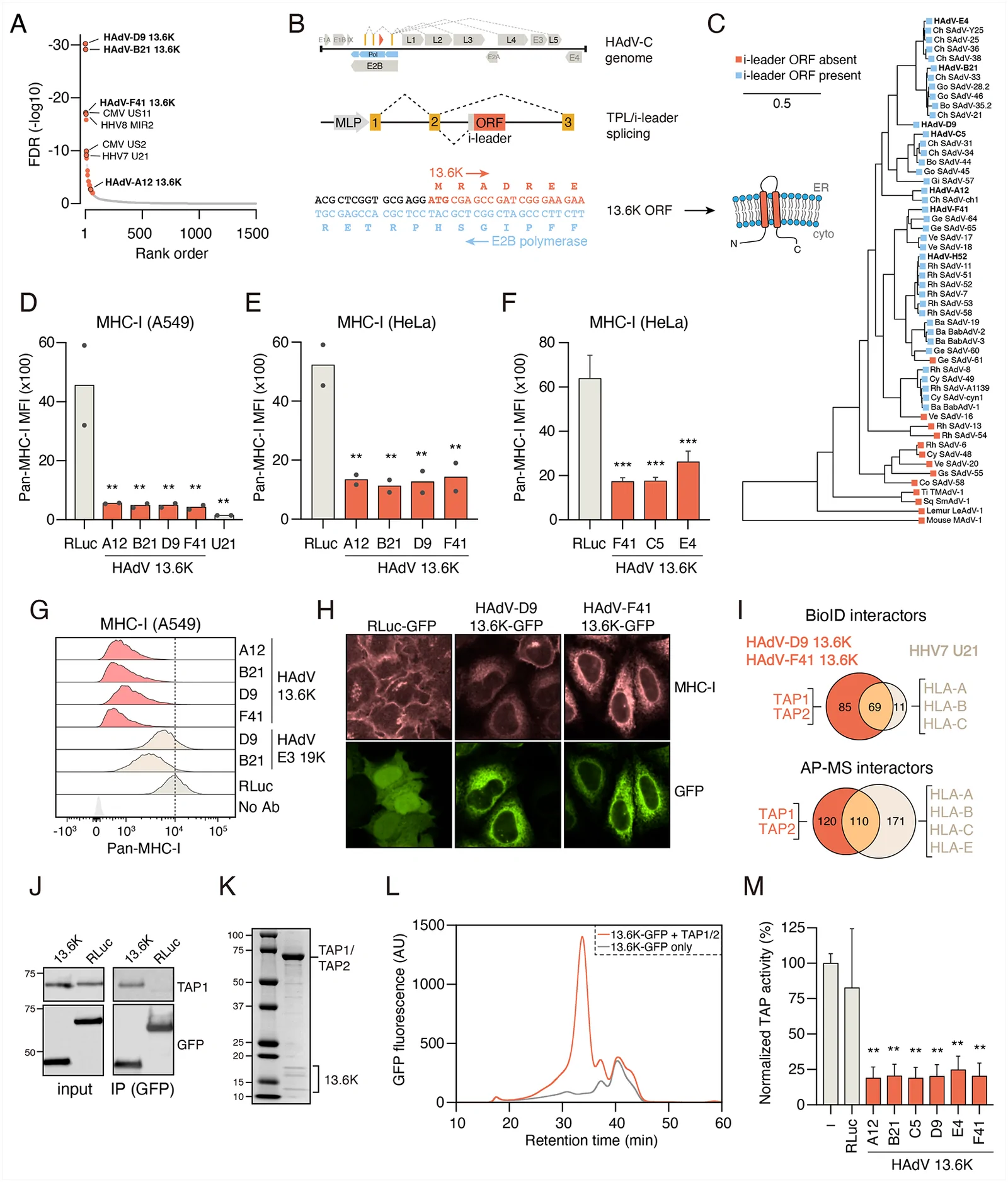
Adenoviral 13.6K/i-leader protein is a recently evolved inhibitor of the TAP transporter. (A) Enrichment ranking plot of eORFs from the MHC-I display screen in A549 cells. Significant hits are highlighted in red. Notable known inhibitors of MHC-I signaling are labeled, with 13.6K clones present in the library highlighted as novel MHC-I regulators. All other eORFs are shown in grey. (B) 13.6K is encoded by the i-leader ORF located between the second and third adenoviral tripartite leader exons, on the opposite strand of the E2B polymerase. (C) Phylogenetic analysis of i-leader ORF presence in primate adenoviruses. Human adenoviruses are represented by a single strain from each clade whereas primate adenoviruses include representatives of 98% sequence identity clusters. Ch, chimpanzee; Bo, bonobo; Go, gorilla; Gi, gibbon; Ge, gelada baboon; Ve, vervet monkey; Rh, rhesus macaque; Ba, baboon; Cy, cynomolgus macaque; Gs, Golden snub-nosed monkey; Co, Colobus monkey; Ti, titi monkey; Sq, squirrel monkey. (D) A549 cells stably expressing dox-inducible GFP-tagged 13.6K proteins or HHV7 U21 were treated with dox for 24 hours followed by flow cytometry analysis of cell surface MHC-I levels. (One-way ANOVA with Dunnett’s comparisons test **padj < 0.01). (E) The same 13.6K-GFP constructs were analyzed in HeLa cells as in (D). (One-way ANOVA with Dunnett’s comparisons test **padj < 0.01). (F) 13.6K-GFP proteins from HAdV-F41 and HAdV-C5 were similarly analyzed in HeLa cells. (One-way ANOVA with Dunnett’s comparisons test ***padj < 0.001, n = 3). (G) Comparison of MHC-I surface levels in A549 cells expressing 13.6K-GFP or the previously known immunoevasin E3 19K-GFP from HAdV-D9 or HAdV-B21. (H) HeLa cells expressing HAdV-D9 or HAdV-F41 13.6K-GFP or Renilla luciferase fused to GFP were stained for MHC-I. The GFP channel shows localization of the GFP-tagged constructs. (I) HAdV-D9 or HAdV-F41 were tagged with miniTurbo-3xFLAG in HeLa cells and analyzed for physical and proximity interactors with BioID and AP-MS, respectively. HHV7 U21 served as a control for an immunoevasin that inhibits surface trafficking of MHC-I molecules. Venn diagrams show the overlap of 13.6K and U21 interactors. (J) HAdV-D9 13.6K-GFP or Renilla luciferase-GFP expressed in HeLa cells was immunoprecipitated with an anti-GFP antibody followed by western blotting for endogenous TAP1 or GFP. (K) HAdV-A12 13.6K-GFP was co-expressed with TAP1-mCherry and TAP2 in HEK293S cells and purified with anti-GFP affinity chromatography and size exclusion chromatography. Top SEC fractions were pooled and run on an SDS-PAGE gel. (L) 13.6K-GFP was transfected into HEK293S TAP1 knockout cells with or without untagged TAP1 and TAP2 followed by fluorescence size exclusion chromatography. (M) GFP-tagged Renilla luciferase or 13.6K from indicated adenoviral serotypes were expressed in HeLa cells and semi-permeabilized followed by a translocation assay of fluorescently labeled TAP substrate peptide into the ER in the presence of ATP and Mg^2+^. Transported peptide was detected by flow cytometry. (One-way ANOVA with Dunnett’s comparisons test **padj < 0.01, n = 3).

Our MHC-I surface display screen identified multiple known regulators of MHC-I display. These included HHV7 U21 characterized above, HCMV US11 and US2 proteins that dislocate MHC-I molecules from the ER to the cytosol for proteasomal degradation,^96,97^ and KSHV MIR2 E3 ligase that ubiquitinates and degrades MHC-I (Figure 6A).^98^ However, the most prominent novel hits were 13.6K proteins from four distinct human adenovirus serotypes HAdV-A12, HAdV-B21, HAdV-D9, and HAdV-F41 (Figure 6A).

The 13.6K protein (also known as i-leader protein) is encoded on the opposite strand of the essential E2B polymerase by an open reading frame located between the second and third exons of the tripartite leader (TPL).^99^ The TPL is a constant 5’ leader sequence for all late adenoviral transcripts (Figure 6B), and some leader splicing events include the intervening leader (i-leader) with the open reading frame encoding 13.6K (Figure 6B).^100^ 13.6K is predicted to have two transmembrane domains with both termini located on the cytoplasmic side (Figure 6B) but its function was unknown. Interestingly, the i-leader ORF is restricted to adenoviruses of Old World monkeys and hominids, as indicated by the absence of a start codon or the presence of in-frame stop codons in the i-leader ORF in other lineages (Figure 6C). Thus, 13.6K represents a recently evolved viral protein with an unknown function.

We validated that 13.6K proteins from the four human adenovirus strains identified in the screen downregulated MHC-I display in A549 and HeLa cells when fused to C-terminal GFP (Figure 6D and 6E). We also cloned the ORF from two additional serotypes (HAdV-C5, HAdV-E4) that were not included in the original screen. Both robustly downregulated surface MHC-I, indicating that this is a conserved function of the protein (Figure 6F). 13.6K also inhibited surface display of β2 microglobulin, a constant subunit of all MHC class I molecules (Figure S6A and S6B). However, HAdV-F41 13.6K expression had no effect on the surface levels of two other plasma membrane proteins EGFR and CD47, suggesting that the effect is specific to MHC-I rather than 13.6K generally interfering with plasma membrane trafficking (Figure S6C). Moreover, 13.6K proteins were significantly more effective in MHC-I downregulation than the previously characterized adenoviral immunoevasin E3 19K from HAdV-B21 or HAdV-D9 (Figure 6G).^101^

The reduction of MHC-I surface levels could be caused by inhibition of membrane trafficking or by MHC-I degradation. We used microscopy to differentiate between these options. In control cells expressing RLuc-GFP, MHC-I was localized to the plasma membrane as expected (Figure 6H). In contrast, in 13.6K-GFP expressing cells, there was no plasma membrane staining of MHC-I. Instead, we detected MHC-I and 13.6K in the endoplasmic reticulum (Figure 6H). Moreover, 13.6K did not affect total MHC-I levels as assessed by western blotting (Figure S6D), indicating that they are ER-associated membrane proteins that inhibit MHC-I membrane trafficking rather than stability.

To understand how 13.6K disrupts antigen presentation, we characterized the interactomes of HAdV-D9 and HAdV-F41 13.6K in HeLa cells with AP-MS and BioID. We tagged them at the C termini with UltraID-3xFLAG and used HHV7 U21 as a positive control for an effector that downregulates MHC-I, and EGFP as a negative control. As expected, HHV7 U21 associated with HLA-A, HLA-B and HLA-C by both BioID and AP-MS, whereas 13.6K proteins did not significantly associate with these proteins (Figure 6I and Table S5). In contrast, HAdV-D9 and HAdV-F41 13.6K associated with the heterodimeric TAP peptide transporter complex subunits TAP1 and TAP2 in both BioID and AP-MS (Figure 6I). Endogenous TAP1 also co-immunoprecipitated with GFP-tagged HAdV-D9 13.6K, validating the interaction (Figure 6J).

The TAP complex transports cytosolic peptides to the ER for loading them onto MHC-I molecules as part of the peptide loading complex (PLC). Many viral effector proteins are known to target TAP to inhibit MHC-I loading and trafficking to the membrane,^102^ making the TAP heterodimer a strong candidate as the cellular target of 13.6K proteins. To assess if 13.6K directly interacts with the TAP complex, we co-expressed HAdV-A12 13.6K-GFP with human TAP1-mCherry and untagged TAP2 in HEK293S GnTi-cells and purified 13.6K-GFP in detergent with a GFP nanobody resin followed by size exclusion chromatography.^102^ SDS-PAGE revealed a single strong band co-purifying with 13.6K, which was confirmed to consist of TAP1 and TAP2 by mass spectrometry, indicating that 13.6K forms a stable complex with the TAP heterodimer (Figures 6K and S6E and Table S5). We further validated this by co-transfecting HAdV-A12 13.6K-GFP with or without untagged TAP1 and TAP2 into TAP1 knockout HEK293T cells and analyzed 13.6K elution profile by fluorescence size exclusion chromatography.^102^ Co-expression of TAP1 and TAP2 significantly shifted the elution profile of 13.6K-GFP to closely match that of uninhibited GFP-tagged TAP (Figure 6L and S6E). The shift was similar to that observed with the well-characterized TAP inhibitor US6 from human cytomegalovirus (Figure S6F–H).^103^ These data further indicate that 13.6K directly interacts with the TAP complex in human cells.

Finally, to test if 13.6K proteins could directly inhibit TAP peptide transporter activity, we expressed EGFP-tagged 13.6K proteins from four serotypes (A12, B21, D9, and F41) in HeLa cells by lentiviral infection and assessed TAP activity with a peptide transporter assay.^104^ We permeabilized cells expressing GFP-tagged 13.6K or Renilla luciferase with a mild detergent and incubated them with a fluorescently labeled TAP substrate peptide RRYQNSTC(AF647)L in the presence of ATP.^105^ In these conditions, TAP can transport the substrate peptide into the ER, which can be detected by flow cytometry. All 13.6K proteins significantly inhibited TAP activity (Figure 6M), establishing that they represent a novel family of recently evolved viral TAP inhibitors.

## DISCUSSION

### The eORFeome as a discovery platform

The eORFeome constitutes a versatile discovery platform that bridges pathogen genomics and functional biology. By combining a comprehensive, sequence-diverse library of almost 4,000 viral, bacterial, and parasitic effector ORFs with barcoded, inducible expression and multiplexed phenotypic screening, this platform enables systematic mapping of effector activities across diverse host pathways. Unlike CRISPR-based perturbations (CRISPR-KO, CRISPRi, and CRISPRa), which rely on the one-dimensional modulation of endogenous gene activity within a single species, the eORFeome assays the functional potential of thousands of exogenous proteins optimized by evolution to rewire host processes in diverse, potentially multidimensional ways. This approach captures mechanisms inaccessible to genetic perturbation of host genes alone and complements CRISPR-based strategies by identifying exogenous regulators that mimic, oppose, or reveal latent host regulatory principles. Thus, eORFs exploit, as *evolved perturbagens*, a wide variety of cellular target proteins and protein-protein interfaces critical for host cell function and central to the modulation of host cell regulatory networks. The modular design of the eORFeome screening platform makes it adaptable to virtually any cellular process that can be measured at population scale.

### General lessons from diverse eORFeome screens

Functionally, the eORFeome screens illuminate general principles of pathogen-host interaction and, more broadly, of protein evolution and function. First, they establish at a systematic scale that entirely unannotated proteins, often with no structural or sequence similarity to known proteins, can execute highly specific and potent cellular functions. Second, many effectors are multifunctional, influencing distinct pathways through separable domains or interfaces, exemplified by HHV7 U21, which employs distinct regions to inhibit both adaptive immunity via suppressing MHC-I antigen presentation and innate immunity by inducing STING degradation. Third, closely related homologs can diverge sharply in activity, such as the high-risk versus low-risk HPV E6 proteins with distinct abilities to inhibit p53. Yet despite this divergent, rapidly evolving sequence space, diverse effectors recurrently converge on a limited number of host pathways and proteins, highlighting evolutionary “hotspots” of host vulnerability and suggesting that the host proteome might impose a finite set of exploitable interfaces.

### Specific findings from diverse eORFeome screens

Among the specific discoveries, our work identifies (i) U14 from HHV6A as a p53 antagonist that sequesters p53 without degrading it, revealing a mode of inactivation analogous to the unrelated SV40 large T antigen; (ii) HHV7 U21 as a dual-function effector that blocks both antigen presentation and innate DNA sensing, via separable structural modules; and (iii) adenoviral 13.6K proteins as a novel family of TAP inhibitors that suppress MHC-I surface expression. The newly uncovered role of 13.6K proteins is a particularly illuminating example of the function and evolution of pathogenic effectors. 13.6K protein is expressed at high levels during infection,^106^ but no function had been identified. The ability of adenoviruses to evade the adaptive immune system has so far been attributed to the E3 19K immunoevasin that has been shown to interact with and inhibit the surface trafficking of MHC-I molecules. However, human adenovirus A, F, and G species lack E3 19K, leading to the suggestion that these species might evade host immunity by other means and targets.^107^ In contrast, 13.6K is present in all HAdV clades, is more abundant than E3 19K during infection,^108^ and is more potent in downregulating MHC-I surface presentation (Figure 6G), suggesting that it might be a more critical immunoevasin for adenoviral fitness. Strikingly, the 13.6K protein emerged during primate adenovirus evolution by a combination of a novel alternative splicing event in the tripartite leader, yielding the i-leader exon, and the acquisition of an ORF in the opposite strand but in the same reading frame as the highly essential E2B polymerase. This remarkable evolutionary path highlights how pathogens can evolve novel functional proteins *de novo* even within exceptionally highly constrained sequences. More generally, our findings not only define new effector classes and mechanisms but also extend the conceptual framework of viral evolution, showing how new ORFs can emerge through splicing innovation and quickly acquire immunomodulatory activity.

### Outlook and vision: a novel way of discovery

Our work defines a new paradigm for protein function discovery, one that treats pathogen effectors from across kingdoms as an evolutionary library of host modulators and functional building blocks, i.e. *evolved perturbagens*. By studying individual eORFs outside their pathogenic contexts, the approach decouples effector biology from disease models, enabling systematic discovery of molecular functions in a controlled and safe setting across evolutionarily unrelated pathogens. This cross-pathogen perspective accelerates annotation of previously uncharacterized proteins and generates foundational data for using eORFs as molecular tools and for training emerging sequence-to-function models. Moreover, it provides an inspiration for engineering minimal domains or synthetic proteins that modulate host pathways with precision for next-generation therapeutics, pathway engineering, and beyond.

In the longer term, coupling large-scale functional genomics with eORF collections that span highly divergent, unannotated protein sequences will provide a direct mapping from sequence to function and thus enable sequence-to-function protein models. Such data-driven modelling will establish a route toward predictive and design-based biology, where the landscape of natural effector mechanisms serves as a training ground for understanding, predicting, and ultimately engineering cellular control.

### Limitations of the Study

This study aims to identify microbial effectors that interfere with host cellular pathways at large scale, which necessitates reductionist experimental design. All effectors were expressed from a standardized inducible promoter, independent of their native expression levels. Moreover, each effector was assayed in isolation, without the broader context of pathogen infection, including potential cofactors, combinatorial interactions, or host responses. As a consequence, the screen is not expected to capture the full spectrum of physiological effector activities and may miss functions that depend on precise expression levels or cooperative interactions between effectors. In addition, the use of a limited number of cell types and pathway-specific assays constrains the detection of context-dependent or cell-type-specific functions. Despite these limitations, the approach displays a low false-positive rate, in that the hits predominantly validate, and provides a systematic resource to prioritize candidate effectors and generate testable hypotheses, which can be further investigated in more complex and physiologically relevant infection models.

## RESOURCE AVAILABILITY

### Lead contact

Requests for further information and resources should be directed to and will be fulfilled by the lead contacts, Mikko Taipale (mikko.taipale@utoronto.ca) or Alexander Stark (stark@starklab.org).

### Materials availability

All plasmids and cell lines generated in this study are available from the authors upon request.

### Data and code availability

All raw sequencing data have been deposited in the Gene Expression Omnibus (GEO). RNA-seq data have been deposited in the SRA under identifier PRJNA1457878. Mass spectrometry datasets have been deposited with the ProteomeXchange Consortium via the MassIVE and PRIDE repositories under identifiers PXD069484, PXD069483, PXD069487, and PXD078568. The code used in this study can be found at https://github.com/vloubiere/git_eORFscreen. Any additional information required to reanalyse the data reported in this paper is available upon reasonable request.

## STAR★METHODS

### EXPERIMENTAL MODEL AND STUDY PARTICIPANT DETAILS

#### Cell Lines

A549 cells (ATCC, CRL-185), HeLa (ATCC, CCL-2), HEK293T (female, ATCC CRL-3216) and Lenti-293T lentiviral packaging cells (female, Clontech, 632180) were cultured in Dulbecco’s modified Eagle medium (Sigma-Aldrich) supplemented with 10% fetal bovine serum, 2 mM L-glutamine (Sigma-Aldrich) and 1× penicillin-streptomycin (Sigma-Aldrich).

U937 cells (gift from Jason Moffat lab) were cultured in RPMI supplemented with 10% FBS.

Cells were passaged every 2 days using TrypLE Express (Thermo Fisher, 12605010) for dissociation and maintained at <70% confluency. All cells were cultured at 37 °C in a humidified incubator with 5% CO_2_. All the cell lines were determined negative for mycoplasma. Cells were used for experiments within 10 passages from thawing.

#### Animals

Animal husbandry, ethical handling of mice, and all animal work were carried out according to the guidelines approved by the Canadian Council on Animal Care and under protocols approved by the Centre for Phenogenomics Animal Care Committee (18–0272H). The animals used in this study were Rosa-CreERT2 (Gt(ROSA)26Sortm1(cre/ERT2)Tyj/J, in C57/Bl6 background)^82^ and FSF-KrasG12D (Krastm1Dsa, in FVBN background),^81^ kindly provided by Dr. Dieter Saur at Technische Universität München, Munich, Germany. Genotyping was performed by PCR using genomic DNA prepared from mouse ear punches. Both male and female mice were used across experimental cohorts. For CreERT2-injected mouse experiments, intragastric injections of approximately 75 mg tamoxifen/kg body weight (a standard dose of 50 μL tamoxifen/corn oil solution) were administered on postnatal days (P) P4 and P5. For histological analyses, lung tissues were harvested at 10 weeks of age, corresponding to 9 weeks post-tamoxifen injection at P4 and P5. For long-term survival experiments, cohorts were continuously monitored starting at 9 weeks of age and tracked for up to 1 year.

### METHOD DETAILS

#### Virus production

Lenti-X cells were co-transfected with lentiviral plasmids, pCMVR8.74 helper (Addgene plasmid no. 22036) and pCMV-VSV-G (Addgene plasmid no. 8454) or pCMV-Eco (Cell Biolabs) envelope plasmids using polyethylenimine (PEI) transfection (MW 25,000, Polysciences) as previously described.^113^ Virus containing supernatant was clarified by centrifugation and filtered through a 0.45 μm filter. Target cells were infected at limiting dilutions in the presence of 4 μg/mL of polybrene.

#### Generation of A549 Cells Expressing the Ecotropic Receptor

To enable screening of the Lenti-eORFeome library in human cells under Biosafety Level-1 (BSL-1) conditions, we engineered the A549 cell line to express the mouse-specific ecotropic receptor, Slc7a1. This strategy renders the cells permissive to mouse ecotropic-pseudotyped lentivirus while ensuring that the viral particles cannot infect receptor-negative human cells, which minimizes risks of vector instability and the generation of replication-competent lentivirus.^114,115^ To achieve this, the lentiviral plasmid pTP36_EcoRec-Zeo was constructed to express Slc7a1 under the control of the PGK promoter, with a downstream internal ribosome entry site (IRES) driving a Zeocin resistance gene. A549 cells were then transduced with VSV-g pseudotyped lentivirus produced from this plasmid. Following selection with Zeocin, a clonal cell line, designated A549-Eco, was established by sorting single cells into 96-well plates using a FACSAria III cell sorter (BD Biosciences).

#### Generation of clonal reporter cell lines

To generate reporter cell lines for NF-κB and p53 signalling, two lentiviral constructs were created. The NF-κB reporter construct contained four tandem copies of an NF-κB response element driving a minimal promoter and a destabilized enhanced green fluorescent protein (eGFP-mODC(d2)). Similarly, the p53 reporter construct contained 13 tandem copies of a p53 RE driving a minimal promoter and mCherry. Both plasmids included a PGK promoter driving a neomycin resistance gene for selection.

Ecotropic pseudotyped lentivirus was produced for each construct and used to transduce A549-Eco cells. Following selection with G418, populations were FACS-purified by sorting single cells with low basal fluorescence (eGFP or mCherry) into 96-well plates to establish clonal lines. Clones were subsequently validated for reporter functionality. The A549-Eco-NF-κB-eGFP clones were treated with 20 ng/mL TNFα, and the clone with the highest eGFP induction was selected. Likewise, A549-Eco-p53-mCherry clones were treated with 2.5 μM Nutlin, and the clone exhibiting the strongest mCherry induction was chosen for subsequent experiments.

#### eORFeome library

Effector open reading frames as Gateway-compatible entry clones were sourced from previously published pathogen-specific ORF collections^35–37,116–118^ or ordered from DNASU Plasmid Repository (Tempe, AZ). Additional clones were from cDNA collections of viral genes^119,120^ that were amplified with PCR and cloned into pDONR221 Gateway entry vector. Salmonella effector ORFs were amplified from Salmonella genomic DNA, provided by John Brumell (SickKids, Toronto). E. coli and C. rodentium effectors were amplified from genomic DNA provided by Lindsay Burns and Samantha Gruenheid (McGill University, Montreal). Toxoplasma effectors were cloned by RT-PCR from Toxoplasma total RNA provided by Sebastian Lourido (Whitehead Institute, Cambridge, MA).

#### Generation of a Coxiella burnetii Gateway library

A library of *Coxiella burnetii* effector sequences was cloned into pDONR221 as previously described^35–37^. Briefly, 147 sequences were selected from the C. burnetii Nine Mile Phase II RSA439 genome based on their homology to eukaryotic domains, known effectors, or direct observation of translocation.^121–123^ Each ORF was PCR amplified using primers with flanking attB sites, with each product including an in-frame ATG-start site and an amber stop codon (F: ggggacaagtttgtacaaaaaagcaggcttcATG…; R: ggggaccactttgtacaagaaagctgggtcATC…). Each was then cloned into the pDONR221-ccdB vector (Invitrogen) using Gateway BP clonase II (Invitrogen) per manufacturer’s instructions and sequence verified.

#### Generation of barcoded backbone

We developed the pCW57.3-TRE3G destination vector from pCW57.1 (Addgene #41393) by replacing the TET promoter with a TRE3G promoter to minimize leakage expression of pathogen effectors in our library.

The library backbone was prepared by digesting the pCW57.3 plasmid with AgeI-HF and SalI-HF (NEB). The digestion products were separated by agarose gel electrophoresis, and the resulting vector backbone and insert template fragments were excised and purified using the Monarch DNA Gel Extraction Kit (NEB). The vector backbone was subjected to two additional rounds of cleanup using the Monarch PCR & DNA Cleanup Kit (NEB).

The insert template was barcoded using a two-step PCR strategy. First, a 50-cycle linear PCR was performed with a forward primer containing a semi-random 30-bp barcode (NNNNNWSWSWSWSWSNNNNNWSWSWSWSWS, where N is any base, S is G or C, and W is A or T; see Table S6 for primer details). This reaction used KAPA HiFi HotStart ReadyMix (Roche) and was followed by a single fill-in cycle with a reverse primer to generate double-stranded DNA. The barcoded insert was then amplified for four cycles using primers that added the necessary overhangs for Gibson assembly. PCR products were purified at each step, first using an Exo-SAP (NEB) treatment followed by the Monarch PCR & DNA Cleanup Kit, and finally with AMPure XP beads (Beckman Coulter).

The barcoded insert was assembled into the prepared vector backbone using an in-house Gibson Assembly Master Mix at a 5-fold molar excess of insert to vector. The reaction was incubated at 50°C for 1 h, and the final assembled library was purified. The library was then transformed into electrocompetent ccdB-resistant MegaX E. coli (homemade) via electroporation. After recovery in SOC medium, the transformants were pooled and expanded in LB medium containing ampicillin and chloramphenicol. Finally, the bacterial culture was harvested, and the plasmid library was purified using a Plasmid Plus Giga/Mega Kit (Qiagen).

#### Gateway cloning of eORFs into barcoded backbone

Entry clones from the eORFeome collection were organized into 18 standardized subpools, each containing ~384 eORFs, and subcloned into the barcoded pCW57.3 (see above). LR recombination reactions were assembled in duplicate using 150 ng of each entry ORF subpool and 150 ng of destination vector with 1 μL Gateway LR Clonase II in a total volume of 5 μL. Reactions were incubated overnight at 25°C in TE buffer. On each of the subsequent two days, an additional 150 ng of destination vector and 1 μL of LR enzyme were added to each reaction, and the volume was adjusted to 5 μL with TE buffer.

LR products were electroporated into NEB 10-beta electrocompetent cells and plated on LB agar containing carbenicillin (100 μg/mL). Plates were incubated overnight at 30°C. Colony counts confirmed a coverage of ~200 fold for each eORF. Colonies were pooled, resuspended in SOC on ice, pelleted, and plasmid DNA was prepared using an endotoxin-free midiprep kit. For downstream lentivirus production, structural and non-structural effectors were maintained as two separate libraries (eORFeome-L1 and eORFeome-L2).

#### Illumina sequencing of the eORF-barcode dictionary

The eORFeome plasmid library (1 μg) was first linearized by digestion with I-SceI (NEB) and subsequently purified using the Monarch PCR & DNA Cleanup Kit (NEB).

To assemble the Tn5 transposome, equimolar amounts of Tn5ME-fw and Tn5MErev oligonucleotides were annealed. The resulting adapters were then incubated with an in-house Tn5 transposase at room temperature for 30 min. The tagmentation reaction was performed by incubating 12 ng of the linearized plasmid library with the assembled Tn5 transposome in a 2x tagmentation buffer at 55°C for 8 min. The reaction was immediately stopped and purified using AMPure XP beads (Beckman Coulter).

The tagmented DNA was size-selected by separating the products on a 2% agarose gel and excising fragments larger than 150 bp. DNA was extracted using the Monarch DNA Gel Extraction Kit. This size-selected library was then used as a template for PCR amplification using KAPA HiFi HotStart ReadyMix (Roche) with a primer pair where one primer was biotinylated.

The biotinylated PCR products were captured on M280 streptavidin beads (Thermo Fisher Scientific). The bead-bound DNA was then used as a template for a final PCR step to add Illumina sequencing adapters. The final library was subjected to two consecutive rounds of gel extraction, isolating fragments between 150 and 300 bp. The library was given a final purification with the Monarch PCR & DNA Cleanup Kit before being submitted for illumina paired-end sequencing.

#### Screens

##### Pooled eORFeome screens

The pooled-eORFeome library was generated by packaging into ecotropic pseudotyped lentivirus through the transfection of Lenti-X cells with PEI. Viral supernatant was subsequently cleared of cellular debris by filtration through a 0.45-μm PES filter. Target cells, including A549-Eco-NF-κB-eGFP, A549-Eco-p53-mCherry, A549-Eco, or U937-Eco lines, were then transduced at a multiplicity of infection (MOI) below 0.2 to ensure single-copy integration while maintaining a library representation of at least 10,000-fold. Twenty-four h post-transduction, cells were selected with 1 μg/mL puromycin until a non-transduced control plate of cells was eliminated, a process that typically required 4–5 days, during which cells were washed twice daily with 1× PBS and received fresh medium. Following selection, eORF expression was induced with 1 μg/mL dox for 24 h.

Screen-specific protocols were then implemented. For the reporter-based screens, the NF-κB activation screen involved collecting a baseline sample of 15 million cells as the input control before the remaining cells were sorted for the top 5% of the GFP-positive population using a BD FACSAria III sorter. Conversely, for the inhibition screens, cells were first treated with either 20 ng/mL TNFα (NF-κB inhibition) or 2.5 μM Nutlin (p53 inhibition) for 16 h. After this treatment, a 15 million cell input sample was collected, and the remaining cells were sorted to isolate the GFP-low or mCherry-low populations (bottom 5%), respectively. For the cGAS-STING inhibition screen, transduced U937 cells were treated with 10 μg/mL cGAMP, resuspended in PBS with 2% FBS containing FVD-780 viability dye (1:1000), and incubated at 4°C for 30 min in the dark. After two washes, the pellet was fixed using the BD Cytofix/Cytoperm^™^ Fixation Buffer, followed by incubation at 4°C for 20 min. Cells were then washed twice with 1x BD Permeabilization Buffer and incubated overnight at 4°C with an anti-IFIT1 primary antibody (Abcam, ab305301, 1:500). The following day, cells were washed twice and incubated with an Alexa Fluor^™^ 647-conjugated goat anti-rabbit secondary antibody (Invitrogen, A-21245; 1:300) for 30 min at 4°C. After two final washes, cells were resuspended in FACS sorting buffer for analysis. In the MHC-I inhibition screen, transduced A549 cells were first incubated with human TruStain FcX (BioLegend, 422302; 1:200) for 10 min at room temperature to block non-specific binding, followed by staining with an APC-conjugated anti-HLA-ABC antibody (BioLegend, 311410; 1:200) for 40 min at 4°C. Following three washes in FACS buffer, the cells were subjected to FACS. For the apoptosis inhibition screen, transduced A549-Eco cells were split post-induction into a control group maintained in dox and a treatment group cultured with both dox and 0.1 μM etoposide. These populations were maintained for 10 days and were split at a 1:4 ratio upon reaching confluency. Similarly, for the cGAMP survival screen, transduced U937 cells were initially treated with 10 μg/mL cGAMP, with the concentration increasing by 1 μg/mL every two days over a 10-day period. To select for strong suppressors, the screen concluded with the addition of 50 μg/mL cGAMP for 2 days. Finally, for every screen, genomic DNA (gDNA) was isolated from all collected cell populations (input, sorted or treated) for subsequent enrichment analysis. All screens were performed in at least two independent biological replicates.

##### DNA Isolation, Barcode PCR/eORFeome PCR, and Sequencing

gDNA was isolated by lysing cell pellets overnight at 55°C in a buffer containing 10 mM Tris-HCl (pH 8.0), 5 mM EDTA, 100 mM NaCl, 1% SDS, and 0.5 mg/mL proteinase K. The lysate was then treated with RNase A (Qiagen) for 2 h at 37°C. Subsequently, gDNA was purified through two sequential extractions with phenol:chloroform:isoamyl alcohol followed by a final extraction with chloroform:isoamyl alcohol.

Sequencing libraries were prepared using one of two strategies, dictated by whether the eORF sequence itself or its associated barcode was amplified for quantification. For the MHC-I and cGAS-STING inhibitor screens, the eORF sequences were directly amplified from gDNA and processed as previously described.^124,125^ For all other screens, eORF-associated barcodes were recovered from gDNA. To this end, the entire gDNA yield from sorted/selected samples and 40 μg of gDNA from input samples were used as templates. To maintain library complexity, 40 individual PCR reactions were performed for each sample. Amplification was carried out for 25 cycles (65°C annealing) using KAPA HiFi HotStart ReadyMix (Roche). The primers were designed to anneal to the Illumina adaptor sequences directly flanking the barcode region. To enable sample demultiplexing, these primers included Illumina i5 and idx index sequences as 5’ overhangs.

Following amplification, all PCR reactions for a given sample were pooled. The pooled amplicons were concentrated and purified using the Monarch PCR & DNA Cleanup Kit (NEB), using six columns for each input sample and one column for each sorted/selected sample. The final purified barcode amplicons were gel-extracted and submitted for next-generation sequencing.

##### RNA-Seq

Uninfected SupT1 and HHV7 infected SupT1 were co-cultured for 7 days at a ratio of 10:1. 1.5 × 10^6^ cells were then harvested from each sample (uninfected, infected, and the co-culture) and were resuspended in 150 μL of DNA/RNA Shield (Zymo) and were submitted to Plasmidsaurus for RNA-seq. Quality of the fastq files was assessed using FastQC v0.12.1. Reads were then quality filtered using fastp v0.24.0 with poly-X tail trimming, 3’ quality-based tail trimming, a minimum Phred quality score of 15, and a minimum length requirement of 50 bp. Quality-filtered reads were aligned to the reference genome using STAR aligner v2.7.11 with non-canonical splice junction removal and output of unmapped reads, followed by coordinate sorting using samtools v1.22.1. PCR and optical duplicates were removed using UMI-based deduplication with UMIcollapse v1.1.0. Alignment quality metrics, strand specificity, and read distribution across genomic features were assessed using RSeQC v5.0.4 and Qualimap v2.3, with results aggregated into a comprehensive quality control report using MultiQC v1.32. Gene-level expression quantification was performed using featureCounts (subread package v2.1.1) with strand-specific counting, multi-mapping read fractional assignment, exons and three prime UTR as the feature identifiers, and grouped by gene_id. Final gene counts were annotated with gene biotype and other metadata extracted from the reference GTF file. Sample-sample correlations for sample-sample heatmap and PCA were calculated on normalized counts (TMM, trimmed mean of M-values) using Pearson correlation. Differential expression was done with edgeR v4.0.16 using standard practice including filtering for low-expressed genes with edgeR::filterByExprwith default values.

##### Hit validations

To validate individual hits, candidate eORFs were subcloned from entry vectors into the barcoded pCW57.3 (for hits from the NF-κB or p53 screens) or pSTV6-eORF-C-GFP-Puro lentiviral plasmid (for hits from the MHC-I or cGAS-STING screens) via Gateway cloning. Lentivirus for each candidate was produced (see above) and used to transduce the appropriate cells in the presence of 4 μg/mL Polybrene. eORF-expressing cell populations were selected with 1 μg/mL puromycin.

For functional validation, each candidate was tested in parallel with and without dox. To identify activators, cells were cultured for 24 h before analysis. To identify inhibitors, cells were cultured for 24 h (with or without dox) and then stimulated for 16 h with the relevant pathway agonist. In all validation experiments, cells expressing a control eORF (mCherry, eGFP or NanoLuc) were included as a negative control. Reporter fluorescence was quantified by flow cytometry using iQue Screener PLUS (Intellicyt).

##### RNA extraction and quantitative PCR

48 h post-induction, U937 cells expressing U21-HHV7 or NanoLuc, alongside uninduced control cells, were harvested. Total RNA was extracted using the RNeasy Mini Kit (Qiagen) according to the manufacturer’s protocol. Subsequently, 1 μg of total RNA was reverse transcribed using the iScript cDNA Synthesis Kit (Bio-Rad). Gene expression levels were normalized to the housekeeping gene *ACTB*. All primer sequences are listed in Table S5.

##### Western Blots

Cells were lysed directly in Laemmli buffer containing 10% β-mercaptoethanol and boiled at 98°C for 5 min. Protein lysates were resolved by SDS-PAGE on 4–15% Mini-PROTEAN TGX Precast Gels (Bio-Rad) and transferred to Immobilon-P PVDF membranes (Merck Millipore) using a wet-transfer system. Membranes were probed with the following primary antibodies: anti-V5-tag (Thermo Fisher, R960–25, 1:1,000), anti-IKKα (Cell Signaling Technology, 11930), anti-IKKβ (Cell Signaling Technology, #8943), anti-Phospho-NF-κB p65 (Ser536) (Cell Signaling Technology, 93H1), anti-IκBα (Cell Signaling Technology, 4814), anti-NF-κB p65 (Cell Signaling Technology, 8242), anti-p53 (Santa Cruz Biotechnology, sc-126), anti-p21 (Santa Cruz Biotechnology, sc-6246), anti-human EGFR (BioLegend, #323123), anti-MHC Class I (Invitrogen, MA5–53005), anti-STING (D2P2F) (Cell Signaling Technology, 13647), anti-Phospho-STING (Ser366) (Cell Signaling Technology, 19781), anti-TBK1/NAK (Cell Signaling Technology, 3013), anti-Phospho-TBK1/NAK (Ser172) (Cell Signaling Technology, 5483), anti-IRF-3 (Cell Signaling Technology, 4302), anti-Phospho-IRF-3 (Ser396) (Cell Signaling Technology, 4947), anti-GFP (Roche, 11814460001; RRID:AB_390913), anti-HA (Sigma-Aldrich, H3663; RRID:AB_262051), anti-H3 (Abcam, ab1791), anti-β-Actin (Cell Signaling Technology, 4967) and anti-HSP90α/β (F-8) (Santa Cruz Biotechnology, sc-13119; RRID:AB_675659) and anti-MHC-I (Invitrogen, MA5–53005).

Detection was performed using HRP-conjugated secondary antibodies (anti-mouse HRP, 7076; anti-rabbit HRP, 074; Cell Signaling Technology, 1:10,000) and Clarity Western ECL Substrate (Bio-Rad). Blots were imaged using a ChemiDoc Imaging System running Image Lab software (Bio-Rad).

##### Protein purification

Purified p53 DBD (residues 94–312) was a kind gift of Daniel Grabarczyk (IMP, Vienna). The U14 coding sequence was cloned into the GoldenBac pGBdest vector by Gibson assembly (in-house), yielding a construct with an N-terminal twin Strep-tag followed by an expression-enhancing linker (EAAAKEAAAKEAAAKEAAAKALEAEAAAKEAAAKEAAAKEAAAKA) and a 3C PreScission protease cleavage site. The plasmid was transformed into DH10 MultiBac cells for bacmid generation, and successful recombinants were identified by blue–white screening.

For baculovirus production, bacmids were transfected into Spodoptera frugiperda (Sf9) cells cultured in ESF921 serum-free medium (Expression Systems). Protein expression was carried out in Trichoplusia ni High-Five insect cells (Thermo Fisher) in 0.5 L cultures at a density of 1.5 × 10^6^ cells/mL, infected with a 1:50 dilution of V1 viral stock and incubated for 3 days at 27°C. Cells were harvested by centrifugation at 600 × g, resuspended in 50 mL of Buffer A (50 mM Tris-HCl pH 8.0, 300 mM NaCl, 1 mM TCEP), flash-frozen, and stored at −70°C.

For purification, thawed cell resuspensions were supplemented with one cOmplete EDTA-free protease inhibitor tablet (Roche) and 50 μL Benzonase (in-house). Cells were lysed using a glass douncer, and the lysate was clarified by centrifugation, followed by PEI precipitation to remove nucleic acids and a second centrifugation step. The clarified lysate was loaded onto a 5 mL StrepTrap HP column (Cytiva) equilibrated with Buffer A using an ÄKTA Pure FPLC system (Cytiva). The column was washed with 10 column volumes of Buffer A, and bound protein was eluted with 2.5 mM desthiobiotin. Fractions containing U14 were pooled, concentrated to 500 μL by ultrafiltration, and further purified by size-exclusion chromatography on a Superdex 200 10/300 GL column (Cytiva) equilibrated in Buffer B (25 mM HEPES pH 7.5, 150 mM NaCl, 0.5 mM TCEP). U14-containing fractions were identified by SDS-PAGE, pooled, concentrated, flash-frozen, and stored at −70°C. Protein concentration was determined by absorbance at 280 nm using an extinction coefficient of 56,270 M^−1^ cm^−1^.

For TAP-13.6K coprecipitation experiments, human TAP1 with a C-terminal mCherry and TAP2 were cloned into a single BacMam baculovirus expression vector to generate pEG TAP-mCherry. The 13.6K sequence from HAdV-A12 was cloned upstream of a GFP connected by a linker containing TEV, SpyTag003, and PreScission Protease cleavage sites to generate pEG HAdV 13.6K-GFP. For whole cell lysate analyses, human TAP1 and TAP2 were cloned without tags into BacMam pEG plasmids. Baculovirus expression vector expressing US6-GFP was prepared previously.^102^

Bacmid produced from pEG TAP-mCherry and pEG HAdV 13.6K-GFP was generated by transforming DH10Bac E. coli cells with the pEG plasmids. Recombinant baculovirus was generated by transfecting Sf9 cells with bacmid using Cellfectin II (Invitrogen). Five days after transfection, P1 baculoviruses were harvested from Sf9 cell media by filtering through a 0.22 μm filter. Baculoviruses were amplified three more times to generate P4 before using for cell transduction.

Proteins were expressed in HEK293S GnTI- cells infected with a baculovirus mixture containing 5% (v/v) of TAP-mCherry and 10% (v/v) 13.6K-GFP at a density of 2 × 10^6^ cells/mL. Cells were induced with 10 mM sodium butyrate 16 h after infection and cultured at 30 °C for another 40 h. Cells were harvested, snap frozen in liquid nitrogen, and stored at −80 °C. For protein purification, cells were thawed and resuspended via glass dounce in lysis buffer containing 50 mM HEPES (pH 6.5 with NaOH), 400 mM NaCl, 2 mM MgCl_2_, 1 mM dithiothreitol (DTT), protease inhibitors (1 μg/mL pepstatin A, 1 μg/mL leupeptin, 1 μg/mL aprotinin, 100 μg/mL soy trypsin inhibitor, 1 mM benzamidine, 1 mM phenylmethylsulfonyl fluoride (PMSF)) and 3 μg/mL DNase I. Cells were lysed in 1% GDN detergent (Anatrace) for 75 minutes at 4 °C before being subjected to ultracentrifugation at 75,000 g for 45 minutes at 4 °C in a Type 45Ti rotor (Beckman). The resulting supernatant was flowed three times through NHS-activated Sepharose 4 Fast Flow resin (GE Healthcare) conjugated with GFP nanobody pre-equilibrated in lysis buffer. The resin was washed with 10 column volumes of Wash Buffer containing 50 mM HEPES (pH 6.5 with NaOH), 400 mM KCl, 2mM MgCl_2_, 1 mM DTT, and 0.02% GDN. To release 13.6K and any bound complexes from the resin-bound GFP, PreScission Protease was added to a final concentration of 0.35 mg/mL and incubated for 3 h at 4 °C. The cleaved protein was eluted with 5 column volumes of wash buffer and collected by passing through Glutathione Sepharose 4B resin (Cytiva) to remove the PreScission Protease. The eluate was then concentrated using a 15 mL Amicon spin concentrator with a 100-kDa molecular weight cutoff membrane (Millipore) and purified by size exclusion chromatography (SEC) using a Superose 6 Increase 10/300 column (GE Healthcare) pre-equilibrated with SEC buffer containing 50 mM HEPES (pH 6.5 with NaOH), 200 mM NaCl, 1 mM DTT, and 0.01% GDN. Collected fractions were pooled and rerun to yield presented chromatogram trace.

##### Analysis of whole-cell lysates

Previously published^102^ HEK293T TAP1 knockout cells were grown in 6-well plates and transiently transfected with pEG plasmids at 37 °C for 48 h. Cells were harvested and spun down before lysing in 200 μL of buffer containing 50 mM HEPES (pH 6.5 with NaOH), 400 mM NaCl, 1% GDN, and protease inhibitors for 45 minutes. Cell lysates were clarified by centrifugation at 75,000 g for 45 minutes at 4 °C. Supernatants were immediately used for analysis by fluorescent size exclusion chromatography (FSEC). FSEC analyses were performed using a Superose 6 10/300 column (GE Healthcare) pre-equilibrated with SEC buffer (20 mM HEPES (pH 6.5 with NaOH), 200 mM NaCl, 0.005% GDN).

##### EMSA

Double-stranded DNA probes were prepared by annealing sense and antisense oligonucleotides. Two probes were used: a p53 response element probe (p53RE; 5’-FAM-CGGGCATGTCCGGGCATGTCCTG-3’) and a scrambled control probe (5’-FAM-CTTACTCGCGGCGAGTGTCCGGG-3’), both 5’-labelled with FAM on the forward strand. Working stocks were diluted to 50 nM in water, and probes were used at a final concentration of 5 nM per reaction.

Binding reactions were performed in 1X EMSA buffer (50 mM Tris-HCl pH 7.5, 10 mM MgCl_2_, 1 mM ATP, 25 μg/mL BSA, 10% glycerol, 10 mM DTT, 200 mM KCl). Purified p53 DBD (residues 94–312; final concentration 400 nM) was first incubated with increasing concentrations of purified U14 for 30 minutes at 4°C. FAM-labelled probe was then added to a final concentration of 5 nM, and reactions were incubated on ice for 90 minutes protected from light. Control reactions included probe alone, p53 alone, and U14 alone.

Samples were resolved on native 6% polyacrylamide gels in 1X Tris-glycine running buffer (25 mM Tris-HCl pH 8.3, 190 mM glycine) at 100V for 80 minutes. FAM-labelled DNA was detected by fluorescence imaging.

##### Immunofluorescence

For U14 experiments, cells expressing empty vector, U14-V5, or the U14 D124R-V5 mutant were seeded in 96-well plates. To induce the expression of the V5-tagged constructs, cells were incubated with dox for 24 h, followed by a 1 h treatment with 1.5 μM Nutlin. Following treatment, cells were seeded in 96-well plates, washed once with PBS, and fixed for 10 min at room temperature with 3.7% formaldehyde in PBS. Following fixation, cells were washed with cold PBS and permeabilized with 0.1% Triton X-100 in PBS for 10 min at room temperature. The cells were then washed three times with PBS. Non-specific binding was blocked by incubating the cells in 5% bovine serum albumin (BSA) in PBS for 30 min at room temperature. Subsequently, cells were incubated overnight at 4°C with primary antibodies diluted in 5% BSA/PBS. The primary antibodies used were mouse anti-p53 (Santa Cruz Biotechnology, sc-126) and rabbit anti-V5-tag (abcam, ab309485) for the detection of V5-tagged U14 or V5-tagged U14 D124R constructs. After primary antibody incubation, cells were washed three times with PBS containing 0.1% Tween-20. Cells were then incubated for 1 h at room temperature in darkness with secondary antibodies diluted 1:1000 in 1% BSA/PBS. The secondary antibodies were goat anti-mouse IgG conjugated to Alexa Fluor^™^ 488 and goat anti-rabbit IgG conjugated to Alexa Fluor^™^ 594. Following secondary antibody incubation, cells were washed three times with PBS. Nuclei were counterstained with DAPI for 5 min at room temperature. Finally, cells were rinsed with PBS before imaging. For quantification, the total number of cells for each cell type was determined by counting DAPI-stained nuclei. The number of cells containing p53 foci (Alexa Fluor^™^ 488) and V5 foci (Alexa Fluor^™^ 594) was then counted. The percentage of cells positive for each type of foci was calculated relative to the total cell count. This analysis was performed for three independent experiments, and the data are presented in graphs as the mean ± standard deviation (SD).

For 13.6K experiments, HeLa cells stably expressing 13.6K-GFP were seeded at 8,000 cells per well (96-well format). The following day, expression of 13.6K was induced using dox. 24h after induction, cells were fixed with 4% paraformaldehyde for 15 min and washed with PBS three times. MHC-I staining was performed using anti-MHC-I fused with AF647 antibody (1:20) for 1 h at RT. Cells were then washed with PBS three times and incubated with Hoechst (1 mg/mL) in 2x SSC for 10 min prior to imaging. Imaging was performed using an Opera PerkinElmer Phenix automated confocal microscope. Images were taken using a 40X water objective. For U14 infection experiments, uninfected SupT1 cells were co-cultured with HHV6B infected SupT1 cells for 7 days at a ratio of 10:1. After 7 days of co-culture, 50,000 cells were harvested and centrifuged at 500 rpm for 5 min. Cells were then washed in PBS twice before being fixed with 4% PFA for 30 min at RT. Cells were then washed twice with PBS. Cells were incubated with p53 antibody (1:800 dilution) in permeabilization buffer for 1 h at RT before being washed twice with PBS. Cells were then incubated with Alexa Fluor 647 Goat anti-Rabbit antibody (1:500 dilution) in permeabilization buffer for 1 h at RT. A 96-well plate was coated with poly-lysine for 20 min before being washed 3 times with PBS. Cells were then washed in PBS twice before being resuspended in 2x SSC with Hoechst (1 mg/mL) and seeded in the coated 96-well plate and centrifuged at 500 rpm for 10 min. Imaging was performed using an Opera Perkin Elmer Phenix automated confocal microscope. Images were taken using a 40x water objective.

##### Cell viability assay

For cell viability assays, U937 cells were seeded into 96-well plates at a density of 2,000 cells per well. The cells were induced with 1 μg/mL dox for 24 h, followed by stimulation with the indicated STING agonists for an additional 24 h. To measure viability, 100 μL of CellTiter-Glo reagent (Promega) was added to the 100 μL of cell culture in each well. The plates were agitated on an orbital shaker for 5 min and then incubated for 5 min at room temperature to stabilize the signal. Luminescence was subsequently measured using a BioTek multimode microplate reader.

##### TAP transporter assay

HeLa cells stably expressing C-terminal eGFP-tagged 13.6K constructs were seeded (100,000 cells per well, 48-well format) in a total volume of 500 μL. The following day, expression of the effectors was induced by adding 1 μg/mL of dox. 48h after induction, the cells were washed with PBS, prior to semi-permeabilization with 0.25 mg/mL saponin in PBS 1x for 15 min. The semi-permeabilization buffer was removed and 100 μL of transport buffer solution of 10 mM ATP, 10 mM MgCl_2_ and 1 nM fluorescence peptide in PBS 1x was added for 15 min. The reaction was stopped by adding 900 μL of PBS 1x supplemented with 20 nM EDTA. The samples were directly analyzed by flow cytometry.

##### U14-V5 AP-MS

For U14 experiments, A549-Eco cells were transduced at a high MOI with lentivirus expressing either U14-V5 or an Empty-V5 control and were subsequently selected with 1 μg/mL puromycin. For each biological replicate (n=3), cells from a confluent 15 cm plate were washed twice with ice-cold PBS and harvested.

Cell pellets were lysed in IP lysis buffer (20 mM HEPES pH 7.3, 150 mM NaCl, 2 mM MgCl_2_, 0.25% NP-40, 10% glycerol, 0.3% Triton X-100) supplemented with cOmplete^™^ EDTA-free Protease Inhibitor Cocktail (Roche). Lysates were incubated on ice for 30 min and sonicated. The lysates were then clarified by centrifugation at 20,000 × g for 5 min at 4°C.

The cleared supernatants were incubated overnight at 4°C with rotation alongside V5-Trap Magnetic Agarose beads (Chromotek) that had been pre-washed with IP lysis buffer. The following day, the beads were washed three times for 10 min each with IP lysis buffer, followed by four 5-minute washes with a no-detergent buffer (20 mM Tris pH 7.5, 130 mM NaCl). The final washed beads were submitted to the Vienna BioCenter Core Facilities (VBCF) Proteomics facility for on-bead digestion and subsequent mass spectrometry analysis.

##### Co-immunoprecipitation

For 13.6K experiments, co-immunoprecipitation experiments were performed using HeLa cells stably expressing 13.6K-GFP constructs. 48 h after induction with dox, the cells were washed with 1x PBS and lysed in NP40 lysis buffer (10 mM Tris/HCl pH 7.5, 150 mM NaCl, 0.5 mM EDTA and 0.5% NP40) with protease inhibitors on ice for 30 min. The lysate was centrifuged at 15,000g for 15 min at 4°C. Proteins were immunoprecipitated using GFP-Trap beads (ProteinTech) at 4°C for 1 h. Beads were then washed 3 times using wash buffer (10 mM Tris/HCl pH 7.5, 150 mM NaCl, 0.5 mM EDTA and 0.05% NP40) to remove non-specific interactions. The immunoprecipitated proteins were eluted from beads by adding loading buffer and heating at 95°C for 5 min. Samples were then loaded on 4%−12% Bis-Tris PAGE and analyzed by Western Blotting on nitrocellulose membranes. Membranes were blotted with anti-GFP (11814460001, Roche, 1:1000) and anti-TAP1 (11114–1-AP, ProteinTech, 1:1000).

For U21 experiments, co-immunoprecipitation experiments were performed using HeLa cells stably expressing U21, U21^Δtail^, U21^NRD^ or U21^4D^ tagged with GFP. 48 h after induction with dox, the cells were washed with 1x PBS and lysed in digitonin lysis buffer (10 mM Tris/HCl pH 7.4, 150 mM NaCl, 0.5 mM EDTA and 0.5% digitonin) with protease inhibitors on ice for 30 min. The lysate was centrifuged at 15,000g for 15 min at 4°C. Proteins were immunoprecipitated using GFP-Trap beads (ProteinTech) at 4°C for 1 h. Beads were then washed 3 times using wash buffer (10 mM Tris/HCl pH 7.5, 150 mM NaCl, 0.5 mM EDTA and 0.05% Digitonin) to remove non-specific interactions. The immunoprecipitated proteins were eluted from beads by adding loading buffer and heating at 95°C for 5 min. Samples were then loaded on 4%−12% Bis-Tris PAGE and analyzed by Western Blotting on nitrocellulose membranes. Membranes were blotted with anti-GFP (11814460001, Roche, 1:1000) and anti-STING (11114–1-AP, ProteinTech, 1:1000).

##### U14 BioID

For the BioID proximity labelling experiment, A549-Eco cells were transduced at a high MOI with lentivirus expressing either U14-TurboID or an Empty-TurboID control and were subsequently selected with 1 μg/mL puromycin. For each biological replicate (n=3), cells from a confluent 15 cm plate were treated with 500 μM biotin for 10 min at 37°C. The cells were then washed twice with ice-cold PBS and harvested.

Cell pellets were lysed in RIPA buffer (50 mM Tris-HCl pH 7.5, 150 mM NaCl, 0.1% SDS, 0.5% sodium deoxycholate, 1% Triton X-100) supplemented with cOmplete^™^ EDTA-free Protease Inhibitor Cocktail (Roche) and 1 mM DTT. After a 1 h incubation at 4°C, lysates were clarified by centrifugation at 18,000 × g for 10 min.

The cleared supernatants were incubated with streptavidin magnetic beads (Dynabeads^™^ M-280 Streptavidin, Thermo Fisher) to capture biotinylated proteins. The beads were then subjected to a stringent series of washes: once with RIPA buffer, once with 2% SDS, once with Buffer 2 (50 mM HEPES pH 7.4, 500 mM NaCl, 1 mM EDTA, 0.1% sodium deoxycholate, 1% Triton X-100), once with Buffer 3 (10 mM Tris-HCl pH 7.5, 1 mM EDTA, 250 mM LiCl, 0.5% sodium deoxycholate, 1% NP-40), and finally, four times with a non-detergent wash buffer (20 mM Tris-HCl, 100 mM NaCl). After the final wash, the beads were flash-frozen in liquid nitrogen and submitted to the proteomics facility for on-bead digestion and mass spectrometry analysis.

##### RavE Whole-cell proteomics

For whole proteome analysis, A549-Eco cells were transduced to express RavE, catalytically inactive RavE (E19A, H21A), or mCherry, and subsequently selected with 1 μg/mL puromycin. For each biological replicate (n=3), cell pellets corresponding to 100 μg of protein were washed three times with PBS and submitted to the proteomics facility for sample preparation using the iST high-complexity whole-cell lysate kit.

Peptides were analyzed by nanoLC-MS/MS using a Vanquish Neo UHPLC system coupled to an Orbitrap Astral mass spectrometer (Thermo Scientific). Peptides were first loaded onto a C18 trap column and subsequently separated on an analytical C18 column (25 cm × 75 μm, 1.7 μm particles) at 50°C using a linear gradient from 2% to 35% acetonitrile over 60 minutes at a flow rate of 300 nL/min. Data were acquired in data-independent acquisition (DIA) mode with full MS scans collected in the Orbitrap (m/z 380–980, resolution 240,000) and fragment ion spectra acquired in the Astral analyzer using 5 Da isolation windows.

Raw data were processed using Spectronaut (v20.3, Biognosys) in directDIA+ mode.^126^ Spectra were searched against the UniProt human reference proteome (release 2026-02-03) supplemented with common contaminants. Trypsin/P specificity with up to two missed cleavages was allowed. Carbamidomethylation of cysteine was set as a fixed modification, while methionine oxidation and protein N-terminal acetylation were considered variable modifications. Results were filtered at a false discovery rate of 1% at the precursor and protein level. Protein quantification was performed using Spectronaut default settings. Data were exported and further processed using the MS2Go pipeline, including mode-based normalization and missing value imputation. Protein abundances were normalized for protein length using iBAQ^127^, and differential expression analysis was performed using limma.^128^

##### Whole-cell proteomics and analysis by Tandem Mass Tagging-based proteomics

For whole proteome analysis, we collected 2 million U937 cells expressing either wild-type U21-HHV7, the truncation mutant U21-HHV7^Δtail^ or RLuc. Cells were lysed with 5% SDS, 50 mM TEAB. Lysates were sonicated for 3 cycles of 5sec on, 3 sec off at 30% amplitude using a Sonicator with 1/8” microtip. 25 μg of protein material was reduced at 20 mM DTT for 10 min at 95C and alkylated with 40 mM iodoacetamide for 30 min in the dark. Samples were brought up to final concentration of 5% SDS and phosphoric acid was added to a final concentration of 1.2%. 165 μl of S-Trap protein binding buffer (90% methanol, 100 mM TEAB) was added to 27.5 μl of acidified lysate. Resulting mixture was passed through the micro column at 4000xg. The micro column was washed 4 times with the S-Trap protein binding buffer. Each sample was digested with 4 μg of trypsin (in 20 μl of 50 mM TEAB) for 1 h at 47C. Prior to elution, 40 μl of 50 mM TEAB pH 8 was added to the column. Peptides were eluted by centrifugation at 4000xg. Peptides were eluted 2 more times with 40 μl 0.2% formic acid and 40 μl of 50% acetonitrile+0.2% formic acid. Eluted peptides were dried down and stored at −40°C. For data-dependent acquisition (DDA) LC-MS/MS, 250 ng protein equivalent of digested peptides were analyzed using a nano-HPLC (High-performance liquid chromatography) coupled to MS. The sample was loaded onto Evotip Pure per manufacturer instructions. Peptides were eluted from the column (cat#: EV-1137, 15cmx150 μm with 1.5 μm beads) with the 30SPD pre-formed acetonitrile gradient generated by an Evosep One system and analyzed on a timsTOF Pro 2. The Evosep was coupled to the timsTOF Pro 2 using a 10 μm diameter emitter tip. The column toaster was set to 40C. The total DIA protocol is 44 min. The MS1 scan had a mass range of 100–1700 Da in dia-PASEF. TIMS settings were accumulation and ramp time of 100 ms, and within the mobility range (1/K0) of 0.6 to 1.6V·s/cm2. Cycle time 2 s. For MS2, 1 mobility window with 2 ramps was used for 32 mass windows, 29.8 Da wide with 5Da mass overlap. The mobility range was from 0.61/K0 to 1.451/K0. This was at a duty cycle of 100% and a ramp rate of 9.52Hz. 1+ ions are excluded from fragmentation using a polygonal filter. The auto calibration was off.

##### 13.6K and U21 UltraID and AP-MS

HAdV 13.6K and HHV7 U21 clones were transferred from the pDONR221 entry vector into a destination vector carrying a C-terminal UltraID-FLAG tag via Gateway recombination. Stable HeLa cell lines expressing the effector-UltraID-FLAG fusion proteins were then generated. Cells were grown to 70% confluence in 15 cm dishes before inducing gene expression with 1 μg/mL dox for 24 h. 50 μM biotin was then added to each plate for 30 min. Cell pellets were collected for downstream UltraID and AP-MS applications.

For biotinylation of UltraID analysis, the cells were treated with 50 μM biotin (Sigma, B4501) for 1 hr, rinsed, and harvested and frozen in PBS buffer. UltraID (n=2) samples were prepared for MS analysis by the NBCC Proteomics Core at the Lunenfeld-Tanenbaum Research Institute (Toronto, Canada). Frozen cell pellets were lysed in 1:4 (cell pellet weight:lysis buffer) using RIPA-lysis buffer (50 mM Tris-HCl (pH 7.5), 150 mM NaCl, 0.1% (w/v) SDS, 1% NP-40, 1 mM MgCl_2_, 1 mM EGTA, 0.5 mM EDTA, 0.4% (w/v) sodium deoxycholate, 1 mM PMSF, and 1x Sigma protease inhibitors). The lysate was sonicated (3 × 5sec, 2 sec off) at 30% amplitude using a 1/8” microtip. 250 units TurboNuclease and 10 μg RNase was added to each sample and incubated, with rotation, at 4°C for 30 min. Additional SDS was added to bring the final concentration to 0.4% SDS. Each sample was mixed well and centrifuged at 14,000rpm for 20 min at 4°C. 200 μl lysate was applied to 20 μl of pre-washed 50% slurry streptavidin beads (Cytiva 17-5113-01), rendered trypsin-resistant, and incubated at 4°C, rotating, for 3 h. After incubation, supernatant was removed and beads were moved to a new tube in RIPA-wash buffer (50 mM Tris-HCl pH 7.4, 150 mM NaCl, 1 mM EDTA, 1% NP-40, 0.1% (w/v) SDS, 0.4% (w/v) sodium deoxycholate). Beads were washed one time with 2% SDS, 50 mM Tris-HCl pH 7.5, two times with RIPA-wash buffer, one time with TNNE-wash buffer (25mM Tris-HCl pH 7.4, 150 mM NaCl, 0.1% NP-40, 1 mM EDTA), and three times with 50 mM ammonium bicarbonate. Proteins on beads were digested with 0.5 μg of trypsin in 50 μl of 50 mM ammonium bicarbonate, overnight at 37°C. 0.25 μg trypsin was added to the peptides and incubated at 37°C for 3 h. Peptides were moved to a new tube, beads were washed with 50 μl water and this wash was combined into the same tube. Formic acid was added to a final concentration of 5%. The solution was centrifuged at 14,000rpm for 5 min, 80% of the solution was moved to a new tube. Peptides were dried down and stored at −40°C until mass spectrometry analysis.

For AP-MS, frozen cell pellets were lysed in 1:4 (cell pellet weight:lysis buffer) using lysis buffer (50 mM HEPES-NaOH pH 8.0, 100 mM KCl, 2mM EDTA, 0.1% NP-40, 10% glycerol, 1 mM DTT, 1 mM PMSF, and 1x Sigma protease inhibitors) for 13.6K and lysis buffer (10 mM Tris-HCl pH 7.4, 150 mM NaCl, 0.5 mM EDTA, 1% Digitonin, 1 mM PMSF and 1x Sigma protease inhibitors) for U21. The lysate was sonicated (3 × 5sec, 2 sec off) at 30% amplitude using a 1/8” microtip. 250 units TurboNuclease and 10 μg RNase was added to each sample and incubated, with rotation, at 4°C for 15 min. Each sample was mixed well and centrifuged at 14,000rpm for 20 min at 4°C. 400 μl lysate was applied to 25 μl of pre-washed 50% slurry anti-FLAG M2 magnetic beads (Sigma-Aldrich M8823) and incubated at 4°C, rotating, for 3 h. After incubation, supernatant was removed and beads were moved to a new tube in lysis buffer, with no PMSF, DTT, or protease inhibitors. Beads were washed one time with FLAG rinsing buffer (20 mM Tris-HCl pH 8.0, 2mM CaCl_2_). The last wash was removed and proteins on beads were digested with 7.5 μl of trypsin (100 ng/μl in 20 mM Tris-HCl pH 8.0), overnight at 37°C. Peptides were moved to a new tube and 2.5 μl trypsin (same concentration) was added to the peptides and incubated at 37°C for 3 h. Formic acid was added to a final concentration of 5%. Peptides were dried down and stored at −40°C until mass spectrometry analysis.

##### 13.6K-GFP purification mass spectrometry

Gel band was washed before undergoing reduction and alkylation, then digested overnight with trypsin. Digestion reactions were stopped with neat FA and extracted into LC-MS vials, prior to being analyzed by LCMS (45 min analytical gradient and separated using a 12 cm pulled emitter column (C18 reversed phase)). The mass spectrometer (Thermo Scientific Lumos) was operated in high res./high mass accuracy mode. Generated data was searched and quantified using ProteomeDiscoverer/Mascot. Data were queried against human database concatenated with contamination database including Trypsin and Lys-C sequences as well as user-submitted sequence.

##### Histology and mouse survival experiment

To induce expression of eGFP, U14, or U14 D124R in vivo, the respective ORFs were cloned into a pLEX306_SV40_Flp_hPGK_DIO-Gateway vector engineered from the pLEX306 backbone (Addgene #41391). Lentivirus (~1.0 × 10^8^ pfu/mL, 5 μL) was delivered via intranasal inhalation to Rosa-CreERT2; FSF-KrasG12D mice at postnatal day 2 (P2). ORF expression was induced by intragastric administration of tamoxifen (~75 mg/kg body weight, 50 μL in corn oil) at P4 and P5.

For H&E staining, mice were euthanized 9 weeks after induction of U14, U14 D124R, or eGFP. Lungs were inflated via intratracheal instillation of 4% paraformaldehyde (PFA) and fixed overnight in 4% PFA. Tissues were transferred to 70% ethanol, embedded in paraffin, and sectioned at 5 μm for H&E staining at the Histology Core Facility, University Health Network (UHN), Toronto, Canada. Tumor area was quantified using NDP.view2 software.

For survival analysis, mice expressing eGFP, U14, or U14 D124R were monitored weekly starting at 9 weeks of age. Body weight was recorded, and animals were euthanized upon reaching humane endpoints, defined as a loss of ≥20% of their maximum body weight.

### QUANTIFICATION AND STATISTICAL ANALYSIS

#### Screen Data analysis

After initial quality checks using fastqc, raw sequencing reads amplified from integrated eORFs after phenotypic selection (screen) or not (input) were trimmed using Trim Galore (v0.6.10) in order to remove adapter sequences and filter out low-quality bases.^129^ Trimmed reads were then aligned to custom indexes that either directly contained eORF sequences (MHC-I and cGAS-STING screens) or a custom dictionary of non-ambiguous barcodes (all other screens) associated to each tested eORF (see Dictionary analysis) using Bowtie2 (v1.3.1).^130^ Reads with low mapping quality – that could not be unambiguously aligned to a unique ORF/Barcode – were discarded using SAMtools (v1.22.1). Finally, reads associated to each ORF/Barcode were counted using the Rsamtools package (v3.21). Resulting matrices were used to identify hits that were significantly enriched after phenotypic selection (screen) compared to unselected/unsorted (input) samples.

For MHC-I and cGAS-STING screens, where integrated eORFs were directly sequenced, hits were identified using DESeq2 (v1.38.3),^110^ and only the eORFs with at least 10 reads in at least two of the compared conditions were considered. For all the other screens, where the abundance of eORFs was inferred from their associated BCs, MAGeCK (v0.5.9)^49^ was used, with the following parameters: --sort-criteria ‘pos’ --norm-method median --remove-zero none. Of note, only the BC/ORFs combinations with at least 3 reads across all screen/input sample pairs were considered, after adding a pseudocount of 1.

The complete scripts detailing the analysis pipeline described above are publicly available at the following GitHub repository: https://github.com/vloubiere/git_eORFscreen.

#### Dictionary analysis

To link eORFs to their corresponding barcodes, we used the eORF-Barcode dictionary which was prepared and sequenced as described in the earlier ‘Illumina sequencing of the eORF-Barcode dictionary’ section. This library consists of DNA fragments where each eORF is positioned at the 5’ end and its associated barcode is at the 3’ end (fragment structure: Tn5_adaptor>eORF>AttB2>22nt_spacer>illuminaFwd>30nt_BC>illuminaRev. Of note, the Tn5 adaptor sequence was used for sequencing, not the illumina Fwd adapter which was only used for screening). Consequently, Read 1 captured the 3’ end of the eORF sequence, while Read 2 captured the barcode sequence, and were used to identify the BCs that were unambiguously assigned to each eORF sequence

First, the 30nt barcode (BC) sequence was extracted from read 2 using cutadapt to trim the illuminaFwd and IlluminaRev adaptor sequences from the 5’ and the 3’ ends (5’ trimming parameters: -g GCTCTTCCGATCT; 3’ trimming parameters: -a AGATCGGAAGAGC -m 25 -M 30). Only barcode sequences that were between 25 and 30nt long and matched the expected sequence pattern ([GC][AT]){4}[GCAT]{5}([GC][AT]){5}[GCAT]{2}) were considered for downstream analyses.

To extract the eORF sequence, three strategies were used: (i) read1 were trimmed using cutadapt (5’ trimming parameters: -g AAAAAAGTTGGCA; 3’ trimming parameters: -a GCCCAACTTTCTT -m 25) or 2 (ii) read 1 were trimmed using Trim Galore^129^ (parameters: --hardtrim5 30) or (iii) read 2 were trimmed using trim_galore (parameters: --hardtrim3 30). eORF sequences were then aligned to the sequences present in the eORFeome library using bowtie2 (v1.3.1, default parameters)^130^ and, for each read ID, only the best alignment (based on mapping quality was retained), and only the reads that were unambiguously aligned (mapping quality ≥ 30) were eventually retained.

Then, confident BC/eORF pairs were retrieved using the read ID, and only the BCs that were supported by at least three read counts and were systematically assigned to the same unique eORF were considered. These barcode sequences were finally used to generate the custom Bowtie2 index used to align the sequencing results from the phenotypic screens.

The complete scripts detailing this analysis is publicly available at the following GitHub repository: https://github.com/vloubiere/git_eORFscreen.

#### Phylogenetic distribution of eORFs

Each eORF was first assigned the taxonomic identifier (TaxID) of its source pathogen from the NCBI Taxonomy database (https://www.ncbi.nlm.nih.gov/taxonomy). These TaxIDs were then used as input to generate a phylogenetic tree based on the NCBI taxonomic hierarchy, which was exported in Newick format. The resulting tree was visualized using the Interactive Tree Of Life (iTOL) web server.^111^

#### Mass Spectrometry Analysis

For data-dependent acquisition (DDA) LC-MS/MS, one-sixteenth of digested peptides were analyzed using a nano-HPLC (High-performance liquid chromatography) coupled to MS. The sample was loaded onto Evotip Pure per manufacturer instructions. Peptides were eluted from the Performance column (cat#: EV-1109, 8cmx150 μm with 1.5 μm beads), heated at 40C) with the 60SPD pre-formed acetonitrile gradient generated by an Evosep One system, and analyzed on a timsTOF Pro 2. The Evosep was coupled to timsTOF Pro 2 using a 20 μm diameter emitter tip. The column toaster was set to 40C. The total DDA protocol is 22 min. The MS1 scan had a mass range of 100–1700Da in PASEF mode. TIMS settings were accumulation and ramp time of 100 ms (with 4 PASEF ramps and active exclusion at 0.4min), and within the mobility range (1/K0) of 0.85 to 1.3V·s/cm2. This was at a cycle time of 0.53s. The target intensity was set to 17,500 and intensity threshold set to 1750. 1+ ions are excluded from fragmentation using a polygonal filter. The auto calibration was off.

For Whole-cell proteomics analysis, MS Data Analysis with Spectronaut, Spectronaut v19 directDIA+ workflow was used to search the data with the Spectronaut generated Human spectral library (Human_PDB_2023). Parameters for the search were default. Differential abundance testing used was unpaired t-test.

For UltraID and AP-MS, Mass spectrometry data generated were stored, searched and analyzed using ProHits laboratory information management system (LIMS) platform. Within ProHits, MSFragger 4.1 was used to search the data using a FASTA database from UP000005640 human Uniprot proteome, no isoforms, with a custom list of contaminants and decoy appended. Acetylated protein N-term and oxidated methionine were set as a variable modifications. Precursor mass tolerance was set to 20ppm on either side. Fragment mass tolerance was set to 20ppm. Enzymatic cleavage was set to trypsin with 2 missed cleavages. MSBooster and Percolator were turned on. Percolator required a minimum probability of 0.5 and did not remove redundant peptides. The target-decoy competition method was used to assign q-values and PEPs. For ProteinProphet, the maximum peptide mass difference was set to 30ppm. When generating the final report, the protein FDR filter was set to 0.01. FDR was estimated by using both filtered PSM and protein lists. Razor peptides were used for protein FDR scoring. All other parameters were default.

#### Homology clusters

To group eORFs by sequence similarity, protein sequences were clustered using MMseqs2 v13.45111^112^ across a range of identity thresholds (0.8, 0.5, 0.3, 0.15 and 0.1).

#### UniProt annotation scores

UniProt annotation scores for eORFs were retrieved from UniProt using their assigned UniProt, CRC64 or RefSeq IDs. In case it matches with more than one entry, the highest score was kept. To note, some viral entries may map to the precursor polyprotein rather than the specific eORF segment. eORFs lacking an annotation score indicate no exact sequence match to any UniProt entry. The human proteome analysis was restricted to reviewed Homo sapiens entries (organism ID: 9606; reviewed:true; n = 20,420).

#### Statistical analysis

GraphPad Prism v10 and RStudio with R v4.2.0 were used for all statistical analysis. Statistical tests are described in figure legends.

#### Clustering of eORF hits

For the clustering, only eORFs significantly enriched in any of the barcoded analyzed with MAGeCK^49^ (FDR ≤ 0.05 and LFC ≥ log2(1.5)) or non-barcoded screens analyzed with DESeq2^110^ (FDR ≤ 0.01 and LFC ≥ 1) were considered. Log fold change (LFC) values were clipped at the 10th and 90th percentiles and LFC of non-significant hits was set to zero. Hits were then clustered using a two-layer self-organizing map using the supersom function from the kohonen R package (v3.0.10) with a 4×5 hexagonal, toroidal grid. The first layer contained the clipped LFC values (user.weight = 1), and the second layer used a binary matrix indicating significant hits (user.weight = 10).

#### Enrichment of homologs

For each cluster of eORF hits defined in the previous section, significantly over-represented protein homology groups (defined as having ≥30% sequence homology using MMseqs2,^112^ as detailed above) were identified using a one-sided Fisher’s exact test (alternative= ‘greater’), with all the other clusters constituting the control group. Of note, only protein homology groups containing ≥ 2 ORFs were considered. Resulting p-values were adjusted for multiple hypothesis testing across all cluster-homology group comparisons using the Benjamini-Hochberg method to control the false discovery rate (FDR).

#### Structural modelling

Predicted three-dimensional structures of the U14-p53 complex and of monomeric RavE were generated using the AlphaFold 3 server (https://alphafoldserver.com/).^131^ The UniProt accession numbers Q69549 (U14) and P04637 (p53) were provided as input for the complex prediction, while Q5ZZ16 was used for RavE. PDB database (https://www.rcsb.org/) was used to retrieve the models of DNA-p53 (PDB ID: 2AC0), SV40 LargeT-p53 (PDB ID:2H1L) and HPV16 E6-p53 (PDB ID: 8GCR). All the resulting structural models were visualized using UCSF ChimeraX (v1.10).^132^

To identify structural homologs, the predicted structure of RavE (amino acids 1–234; UniProt: Q5ZZ16) was used as a query to search the PDB100 database (release 20240101) using Foldseek.^59^ The search was taxonomically restricted to Bacteria.

## Supplementary Material

1

2 TableS1. Clones present in the eORFeome 1.0 collection, related to Figure 1.

3 TableS2. MAGeCK and DESeq2 outputs from eORFeome screens, related to Figure 2–6.

4 TableS3. Functional cluster assignments for eORF screen hits, related to Figure 3.

5 TableS4. AP-MS and BioID experiment result tables with U14, related to Figure 4.

6 TableS5. AP-MS and BioID experiment result tables with 13.6K and U21, related to Figure 5 and 6.

7 TableS6. Oligonucleotides, related to Figure 5 and STAR Methods.

Figures S1–S6, Tables S1–S6

## Figures and Tables

**Table:** KEY RESOURCES TABLE

REAGENT or RESOURCE	SOURCE	IDENTIFIER
Antibodies		
Alexa Fluor^™^ 647 anti-human EGFR Antibody	BioLegend	352917
Alexa Fluor^™^ 647 anti-human HLA-A,B,C Antibody	BioLegend	311414
Anti-IFIT1 antibody [EPR27276–63]	Abcam	ab305301
APC Annexin V	BioLegend	640920
APC anti-human CD47 Antibody	BioLegend	323123
APC anti-human HLA-A,B,C, Clone W6/32	BioLegend	311410; RRID: AB_314879
APC anti-human β2-microglobulin	BioLegend	395712
GAPDH	BioLegend	607904
Goat anti-Mouse IgG Alexa Fluor^™^ 488	Invitrogen	A32723
Goat anti-Rabbit IgG (H+L) Highly Cross-Adsorbed Secondary Antibody, Alexa Fluor^™^ 647	Invitrogen	A-21245
Goat anti-Rabbit IgG Alexa Fluor^™^ 594	Invitrogen	A-11012
H3	Abcam	ab1791
Hoechst 33342	Sigma-Aldrich	B2261
Human TruStain FcX	BioLegend	422302; RRID: AB_2818986
IKK	Cell Signaling Technology	8943
IKKα	Cell Signaling Technology	11930
IRF-3 Antibody	Cell Signaling Technology	4302
IκBα	Cell Signaling Technology	4814
MHC Class I Recombinant Rabbit Monoclonal Antibody	Invitrogen	MA5–53005
Monoclonal ANTI-FLAG(R) M2-Peroxidase (HRP) antibody	Sigma-Aldrich	A8592, RRID:AB_439702
Monoclonal Anti-HA	Sigma-Aldrich	H3663; RRID:AB_262051
Mouse Anti-GFP	Roche	11814460001; RRID:AB_390913
Mouse Anti-HSP90alpha/beta (F-8)	Santa Cruz Biotechnology	sc-13119, RRID:AB_675659
NF-κB p65	Cell Signaling Technology	8242
p21	Santa Cruz Biotechnology	sc-6246
p53	Santa Cruz Biotechnology	sc-126
Phospho-IRF-3 (Ser396)	Cell Signaling Technology	4947
Phospho-NF-κB p65 (Ser536)	Cell Signaling Technology	93H1
Phospho-STING (Ser366)	Cell Signaling Technology	19781
Phospho-TBK1/NAK (Ser172)	Cell Signaling Technology	5483
RIPK1	ProteinTech	9932–1-AP
STING (D2P2F) Rabbit mAb	Cell Signaling Technology	13647
TAP-1 antibody	ProteinTech	11114–1-AP
TBK1/NAK Antibody	Cell Signaling Technology	3013
V5-tag (mouse)	Thermo Fisher	R960–25
V5-tag (rabbit)	abcam	ab309485
β-Actin Antibody	Cell Signaling Technology	4967
Bacterial and virus strains		
ccdB-resistant MegaX *E. coli*	homemade	N/A
DH5α competent Cells	homemade	N/A
DH10 MultiBac cells	homemade	N/A
MegaX *E. coli*	homemade	N/A
NEB^®^ 10-beta Electrocompetent *E. coli*	NEB	C3020K
Stbl3^™^ Chemically Competent *E. coli*	homemade	N/A
Chemicals, peptides, and recombinant proteins		
2’,3’-cGAMP sodium	MCE	HY-100564A
Bafilomycin A1	InvivoGen	inh-bfa
Benzonase	homemade	N/A
Chloroquine	InvivoGen	tlrl-chq-4
Clarity Western ECL Substrate	Bio-Rad	#1705061
DAPI	Sigma-Aldrich	D9542–1 MG
Desthiobiotin	Scientific Lab Supplies	D1411
diABZI (compound 3)	InvivoGen	tlrl-diabzi-2
Dimethyl sulfoxide (DMSO)	Fisher	D128–500
DMEM	homemade	N/A
Doxycycline (dox)	Sigma-Aldrich	D9891
Fetal Bovine Serum	Gibco	S0615
L-glutamine	Gibco	25030081
Laemmli SDS sample buffer, reducing (4X)	Thermo Fisher	J60015.AC
Lenti-X concentrator	Takara	631232
Lipofectamine 3000 Transfection Reagent	Invitrogen	L3000008
MEM Non-Essential Amino Acids Solution (100X)	Thermo Fisher	11140035
Opti-MEM I Reduced Serum Medium	Gibco	31985062
p53 DBD (residues 94–312)	Gift from Daniel Grabarczyk (IMP Vienna)	N/A
Penicillin-streptomycin	Gibco	15140122
Protease Inhibitor Cocktail	Roche	11836153001
Puromycin Dihydrochloride	Gibco	A1113803
Recombinant human IL-1β protein	Invivogen	rcyc-hil1b
Red ANTI-FLAG^®^ M2 Affinity Gel	Sigma-Aldrich	F2426, RRID: AB_2616449
Roti-Phenol/Chloroform/Isoamyl alcohol	Thermo Fisher	FD1464
Staurosporine	Sigma-Aldrich	569397
TNF alpha	Bio-Techne	210-TA-005
Trypan Blue Stain (0.4%)	Invitrogen	T10282
Critical commercial assays		
Dynabeads M-280 Protein G	Thermo Fisher	10003D
GFP-Trap Magnetic Agarose beads	Chromotek	gtma
iTaq^™^ Universal SYBR^®^ Green One-Step Kit	Bio-rad	1725150
KAPA HiFi HotStart ReadyMix	Roche	KK2602
Monarch Spin DNA Gel Extraction Kit	NEB	T1120
Monarch Spin PCR & DNA Cleanup	NEB	T1135
QIAamp DNA Mini Kit	Qiagen	51304
RNeasy Plus Mini Kit	Qiagen	74136
V5-Trap Magnetic Agarose beads	Chromotek	v5tma
Experimental models: Cell lines		
A549	ATCC	CRL-185
HEK293T (female) parental	ATCC	CRL-3216
HeLa	ATCC	CCL-2
LentiX, female	Clontech	632180
LLC1	ATCC	CRL-1642
*Spodoptera frugiperda* Sf9 cells	Expression Systems	94–001F
Trichoplusia ni High-Five insect cells	Thermo Fisher	B85502
U937	Jason Moffat Lab	CRL-1593.2
Experimental models: Organisms/strains		
FSF-KrasG12D (Krastm1Dsa)	Dieter Saur Lab	N/A
Rosa-CreERT2 (Gt(ROSA)26Sortm1(cre/ERT2)Tyj/J)	Dieter Saur Lab	N/A
Deposited data		
MS data of BioID and AP-MS	This manuscript	[To be requested by reviewers]
Raw eORFeome screen sequencing files	This manuscript	[To be upload to GEO]
RNA-Seq data	This manuscript	SRA identifier: PRJNA1457878
Oligonucleotides		
PCR primer sequences, see Table S6	This manuscript	N/A
Real-time PCR primer sequences, see Table S6	This manuscript	N/A
sgRNA and shRNA sequences, see Table S6	This manuscript	N/A
Recombinant DNA		
pCMV-Eco	Cell Biolabs	RV-112
pCMV-VSV-G	Addgene	#8454
pCMVR8.74	Addgene	#22036
pGBdest-U14	This manuscript	N/A
pPGK_EcoRec-Zeo	This manuscript	N/A
pPGK-Slc7a1-ZeoR	This manuscript	N/A
psPAX2	Addgene	#12260
pSTV6-TetO-ccdB-eGFP	Anne-Claude Gingras Lab	N/A
pTRE_TurboID_FLAG	This manuscript	N/A
pVSVG	Addgene	#12259
Software and algorithms		
Adobe Illustrator	Adobe	2021
Clustal-Omega	Sievers et al.^109^	https://www.ebi.ac.uk/jdispatcher/msa/clustalo
Custom analyses	This manuscript	https://github.com/vloubiere/git_eORFscreen
DESeq2 version 1.48.2	Love et al.^110^	https://bioconductor.org/packages/release/bioc/html/DESeq2.html
FlowJo	BD Biosciences	Version 10.8
Foldseek release 10–941cd33	van Kempen et al.^59^	https://github.com/steineggerlab/foldseek
GraphPad Prism	GraphPad	Version 10.0
ImageJ	NIH	Version 1.49v
iTOL version 7 web server	Letunic and Bork^111^	https://itol.embl.de/
MMseqs2	Steinegger and Soding^112^	https://github.com/soedinglab/MMseqs2
Other		
Thermal Cycler C1000	Bio-Rad	N/A
FACSAria III cell sorter (BD Biosciences)	BD Biosciences	N/A
iQue Screener PLUS	Intellicyt	N/A
StrepTrap HP column	Cytiva	N/A
Superdex 200 10/300 GL column	Cytiva	N/A
AKTA Pure FPLC system	Cytiva	N/A

## HIGHLIGHTS

- The eORFeome: a collection of ~4,000 viral, bacterial, and parasitic effector ORFs
- Pooled lentiviral screens across five core pathways reveals new effector functions
- HHV6A U14 sequesters p53 into nuclear foci and blocks DNA binding
- Adenoviral 13.6K is a de novo evolved TAP inhibitor that suppresses MHC-I display
