# Supplementary material for: Systematic Discovery of Pathogen Effector Functions across Human Pathogens and Pathways
Source: Cell. Author manuscript; Available in PMC 2026 Jul 17. (PMC13378530; doi:10.1016/j.cell.2026.06.017)
Supplement: 1 [file NIHMS2193300-supplement-1.pdf]

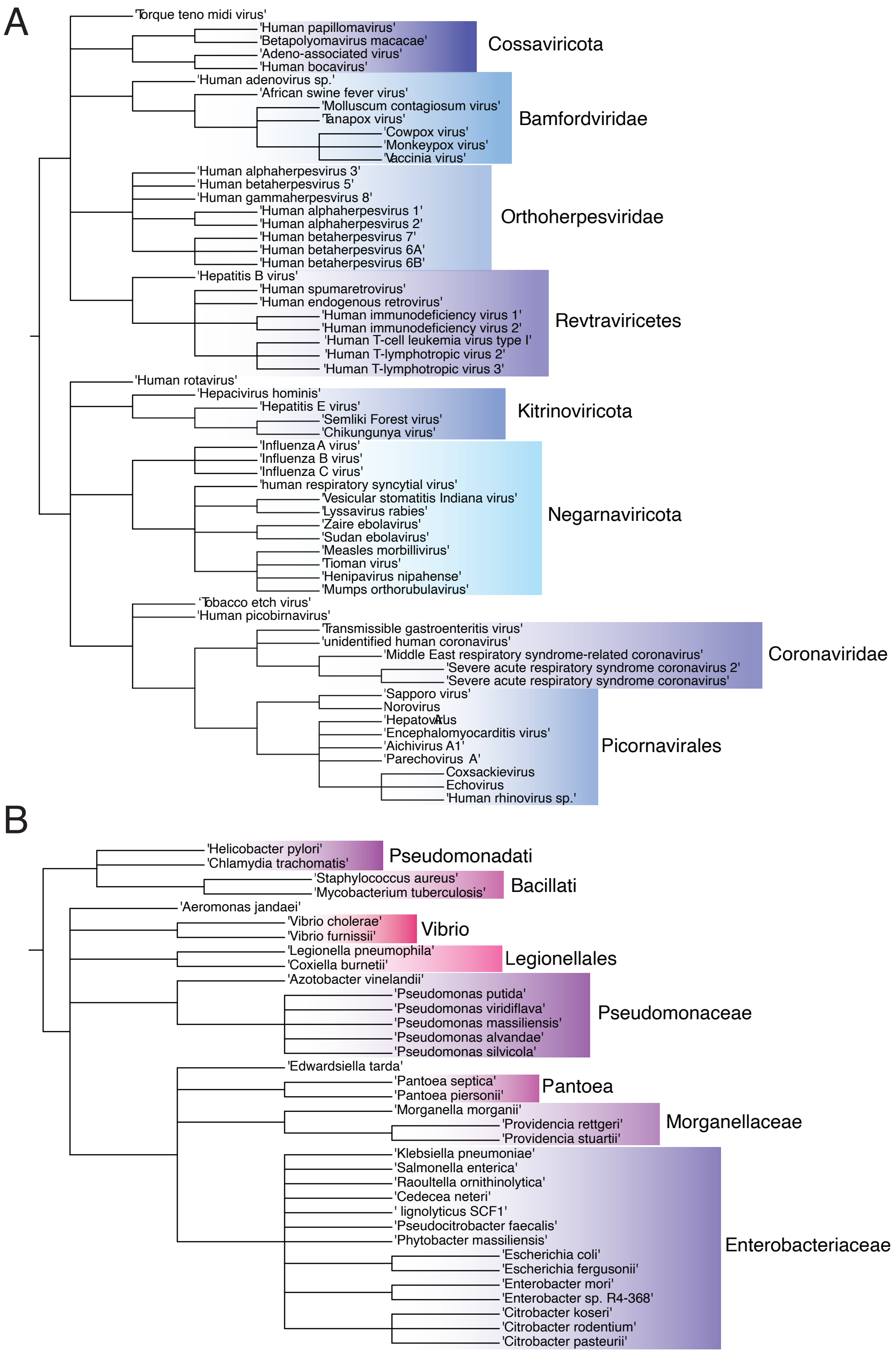

A
'Torque teno midi virus'
'Human papillomavirus'
'Betapolyomavirus macacae'
'Adeno-associated virus'
'Human bocavirus'
Cossaviricota
'Human adenovirus sp.'
'African swine fever virus'
'Molluscum contagiosum virus'
'Tanapox virus'
'Cowpox virus'
'Monkeypox virus'
'Vaccinia virus'
Bamfordviridae
'Human alphaherpesvirus 3'
'Human betaherpesvirus 5'
'Human gammaherpesvirus 8'
'Human alphaherpesvirus 1'
'Human alphaherpesvirus 2'
'Human betaherpesvirus 7'
'Human betaherpesvirus 6A'
'Human betaherpesvirus 6B'
Orthoherpesviridae
'Hepatitis B virus'
'Human spumaretrovirus'
'Human endogenous retrovirus'
'Human immunodeficiency virus 1'
'Human immunodeficiency virus 2'
'Human T-cell leukemia virus type I'
'Human T-lymphotropic virus 2'
'Human T-lymphotropic virus 3'
Revtraviricetes
'Human rotavirus'
'Hepacivirus hominis'
'Hepatitis E virus'
'Semliki Forest virus'
'Chikungunya virus'
Kitrinoviricota
'Influenza A virus'
'Influenza B virus'
'Influenza C virus'
'human respiratory syncytial virus'
'Vesicular stomatitis Indiana virus'
'Lyssavirus rabies'
'Zaire ebolavirus'
'Sudan ebolavirus'
'Measles morbillivirus'
'Tioman virus'
'Henipavirus nipahense'
'Mumps orthorubulavirus'
Negarnaviricota
'Tobacco etch virus'
'Human picobirnavirus'
'Transmissible gastroenteritis virus'
'unidentified human coronavirus'
'Middle East respiratory syndrome-related coronavirus'
'Severe acute respiratory syndrome coronavirus 2'
'Severe acute respiratory syndrome coronavirus'
Coronaviridae
'Sapporo virus'
Norovirus
'Hepatovirus A'
'Encephalomyocarditis virus'
'Aichivirus A1'
'Parechovirus A'
Coxsackievirus
Echovirus
'Human rhinovirus sp.'
Picornavirales
B
'Helicobacter pylori'
'Chlamydia trachomatis'
Pseudomonadati
'Staphylococcus aureus'
'Mycobacterium tuberculosis'
Bacillati
'Aeromonas jandaei'
'Vibrio cholerae'
'Vibrio furnissii'
Vibrio
'Legionella pneumophila'
'Coxiella burnetii'
Legionellales
'Azotobacter vinelandii'
'Pseudomonas putida'
'Pseudomonas viridiflava'
'Pseudomonas massiliensis'
'Pseudomonas alvandae'
'Pseudomonas silvicola'
Pseudomonaceae
'Edwardsiella tarda'
'Pantoea septica'
'Pantoea piersonii'
Pantoea
'Morganella morganii'
'Providencia rettgeri'
'Providencia stuartii'
Morganellaceae
'Klebsiella pneumoniae'
'Salmonella enterica'
'Raoultella ornithinolytica'
'Cedecea neteri'
' lignolyticus SCF1'
'Pseudocitrobacter faecalis'
'Phytobacter massiliensis'
'Escherichia coli'
'Escherichia fergusonii'
'Enterobacter mori'
'Enterobacter sp. R4-368'
'Citrobacter koseri'
'Citrobacter rodentium'
'Citrobacter pasteurii'
Enterobacteriaceae

Supplementary Figure 1. Evolutionary relationships of eORFs in the eORFeome library, related to Figure 1. Phylogenetic tree of the viral (A) or bacterial (B) eORFs present in the eORFeome library.

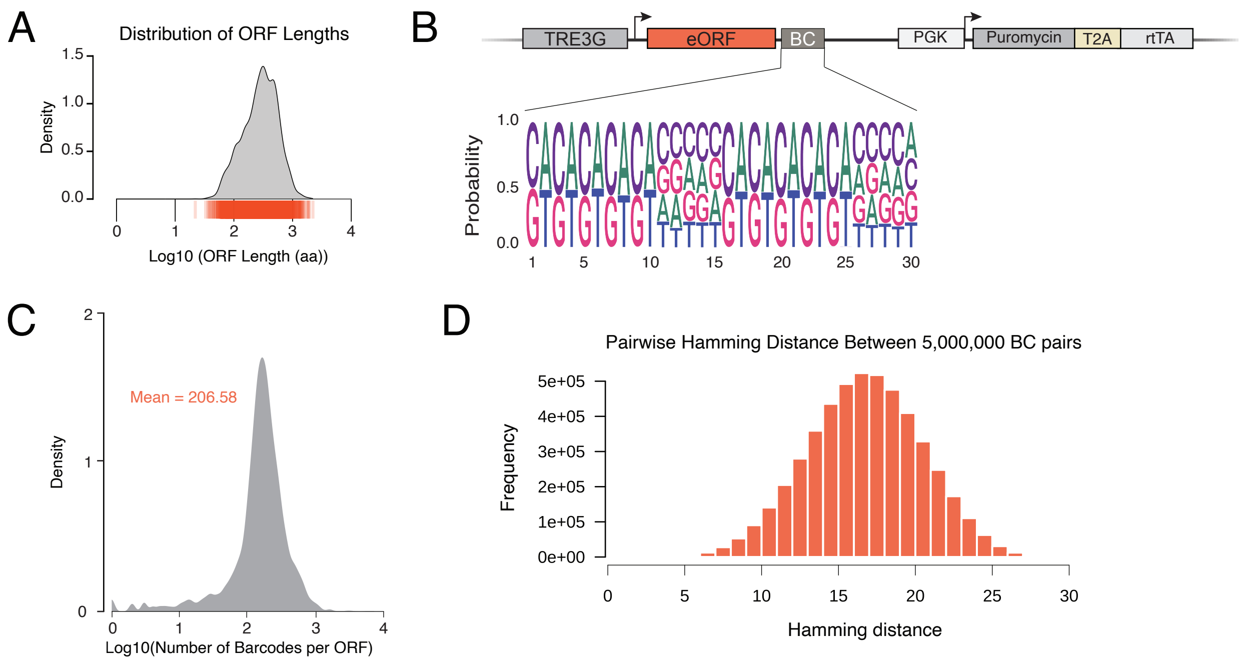

A
Distribution of ORF Lengths
Density
1.5
1.0
0.5
0.0
0
1
2
3
4
Log10 (ORF Length (aa))
B
TRE3G
eORF
BC
PGK
Puromycin
T2A
rtTA
Probability
1.0
0.5
0.0
1
5
10
15
20
25
30
C
Density
2
1
0
Mean = 206.58
0
1
2
3
4
Log10(Number of Barcodes per ORF)
D
Pairwise Hamming Distance Between 5,000,000 BC pairs
Frequency
5e+05
4e+05
3e+05
2e+05
1e+05
0e+00
0
5
10
15
20
25
30
Hamming distance

Supplementary Figure 2. Characterization of the eORFeome library, related to Figure 1. (A) Distribution of eORF lengths. The density plot is based on the Log10-transformed length (in amino acids) of each ORF in the library. The underlying orange lines marks the position of each individual eORF. (B) Top: Scheme of the lentiviral construct, detailing the arrangement of its key components: a dox-inducible TRE3G promoter drives the expression of an eORF, which is followed by a unique barcode (BC). Downstream of this, a constitutive promoter ensures the expression of both puromycin for cell selection and rtTA, which is essential for the dox-inducible activation of the TRE3G promoter. Bottom: position weight matrix that describes the semi-random nature of the 30-nucleotide barcode (BC), which consists of the pattern [(SW)×5 + N×5] repeated twice, where S represents G or C, W represents A or T, and N represents any nucleotide. (C) Density plot of the Log10 distribution of the number of barcodes per ORF, with a mean of 206.58 BC per ORF. (D) Histogram of the Hamming distance distribution, where the frequency (y-axis) of a given Hamming distance (x-axis) was calculated from 5 million randomly sampled barcode pairs from the library.

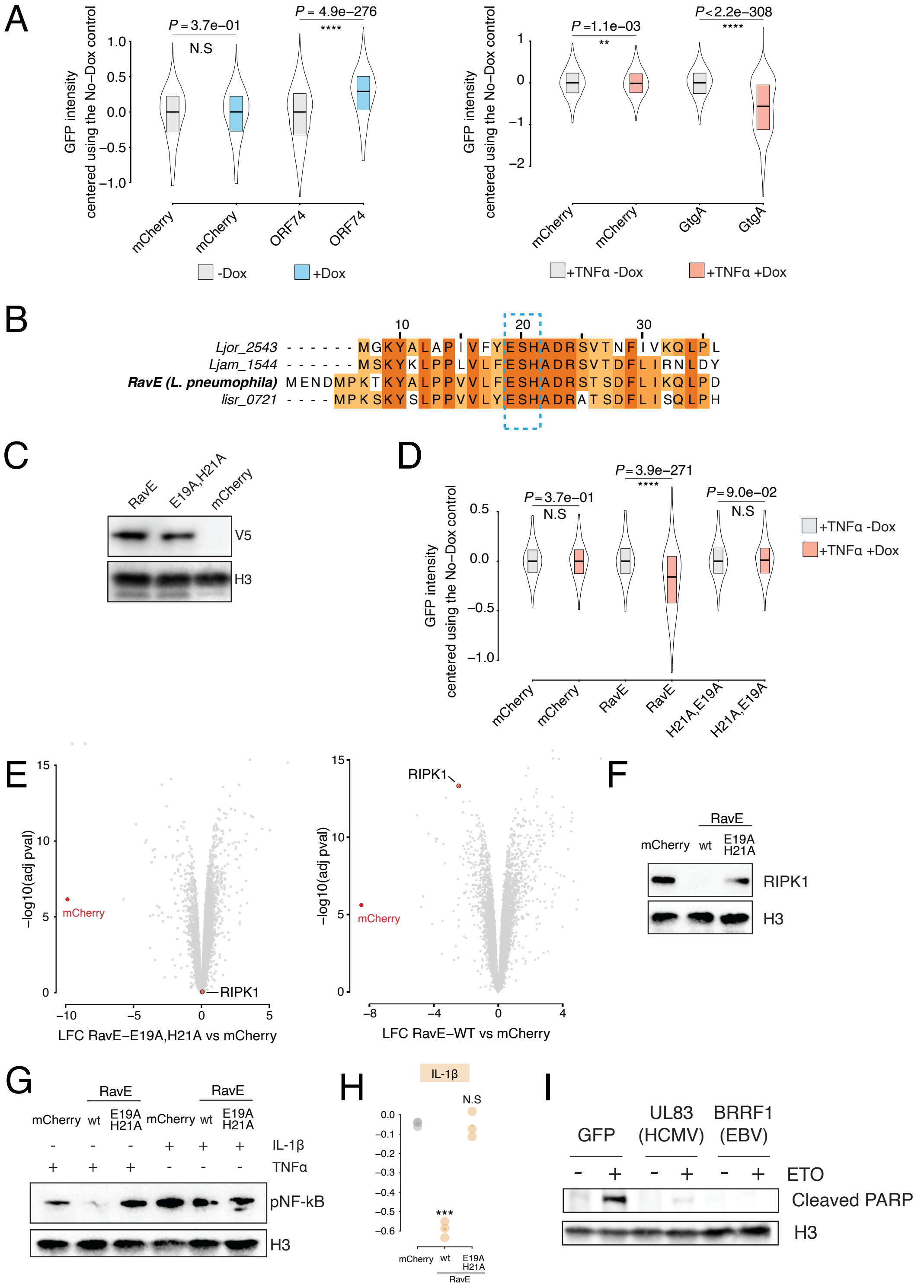

A
GFP intensity centered using the No−Dox control
P = 3.7e−01
N.S
P = 4.9e−276
****
mCherry
ORF74
-Dox
+Dox
P =1.1e−03
**
P < 2.2e−308
****
GtgA
+TNFα -Dox
+TNFα +Dox
B
Ljor_2543
Ljam_1544
RavE (L. pneumophila)
lisr_0721
C
RavE
E19A,H21A
mCherry
V5
H3
D
P = 3.7e−01
P = 3.9e−271
P = 9.0e−02
H21A,E19A
E
−log10(adj pval)
RIPK1
LFC RavE−E19A,H21A vs mCherry
LFC RavE−WT vs mCherry
F
wt
E19A H21A
G
IL-1β
TNFα
pNF-kB
H
***
I
GFP
UL83 (HCMV)
BRRF1 (EBV)
ETO
Cleaved PARP

Supplementary Figure 3. Validation of eORF hits from NF-κB Screens, related to Figure 2 and Figure 3. (A) Boxplots showing representative examples of the single-cell GFP fluorescence distribution, complementing the median-based analysis presented in Figure 2B. ORF expression was induced with dox (blue or orange) or left untreated (grey) and where specified, cells were treated with 20 ng/mL TNFα for 16 hours to stimulate NF-κB activity. The y-axis represents the Logicle-transformed GFP fluorescence, centred to the median of the corresponding no-dox control population. Boxplots display the median and interquartile range. mCherry serves as the negative control. Statistical significance was determined by comparing dox-treated versus untreated samples for each ORF using multiple paired t-tests. (B) Multiple sequence alignment of four RavE homologs. Conserved residues are coloured in orange. (C) Western blot analysis of cells expressing V5-tagged RavE, V5-tagged RavE E19A,H21A mutant, or an mCherry control. Lysates were immunoblotted with an anti-V5 antibody to confirm that the mutant protein is stably expressed. H3 served as a loading control. (D) Boxplots showing single-cell GFP fluorescence distributions, generated using the same analysis method described in (A). These data complement the median-based analysis presented in Figure 2J. (E) Volcano plots of whole-cell mass spectrometry data. The left panel shows the comparison between RavE (E19A, H21A) mutant and the mCherry control, while the right panel shows RavE WT versus mCherry. RIP is highlighted in orange. (F) Western blot analysis of RIP protein levels in cells expressing RavE WT, RavE (E19A, H21A) mutant, or mCherry. (G) Western blot analysis of cell lysates from the three cell lines (RavE WT, RavE E19A,H21A mutant, and mCherry control) following stimulation with either IL-1β or TNFα for 30 minutes. Blots were probed for pNF-κB to assess pathway activation, with Histone H3 (H3) serving as a loading control. (H) Comparison of the inhibitory activity of wild-type RavE and RavE (H21A,E19A) mutant following pathway activation with IL-1β. Statistical significance was determined by comparing each ORF to the mCherry control using multiple paired t-tests. (I) Western blot analysis of cells expressing GFP (control), UL83 from HCMV, or BRRF1 from EBV, either left untreated or treated with 50 μM etoposide (ETO) for 24 hrs. Lysates were immunoblotted for Cleaved PARP to assess apoptotic activation, with H3 serving as a loading control. ****padj < 0.0001, ***padj < 0.001, **padj < 0.01, *padj < 0.05; N.S, not significant.

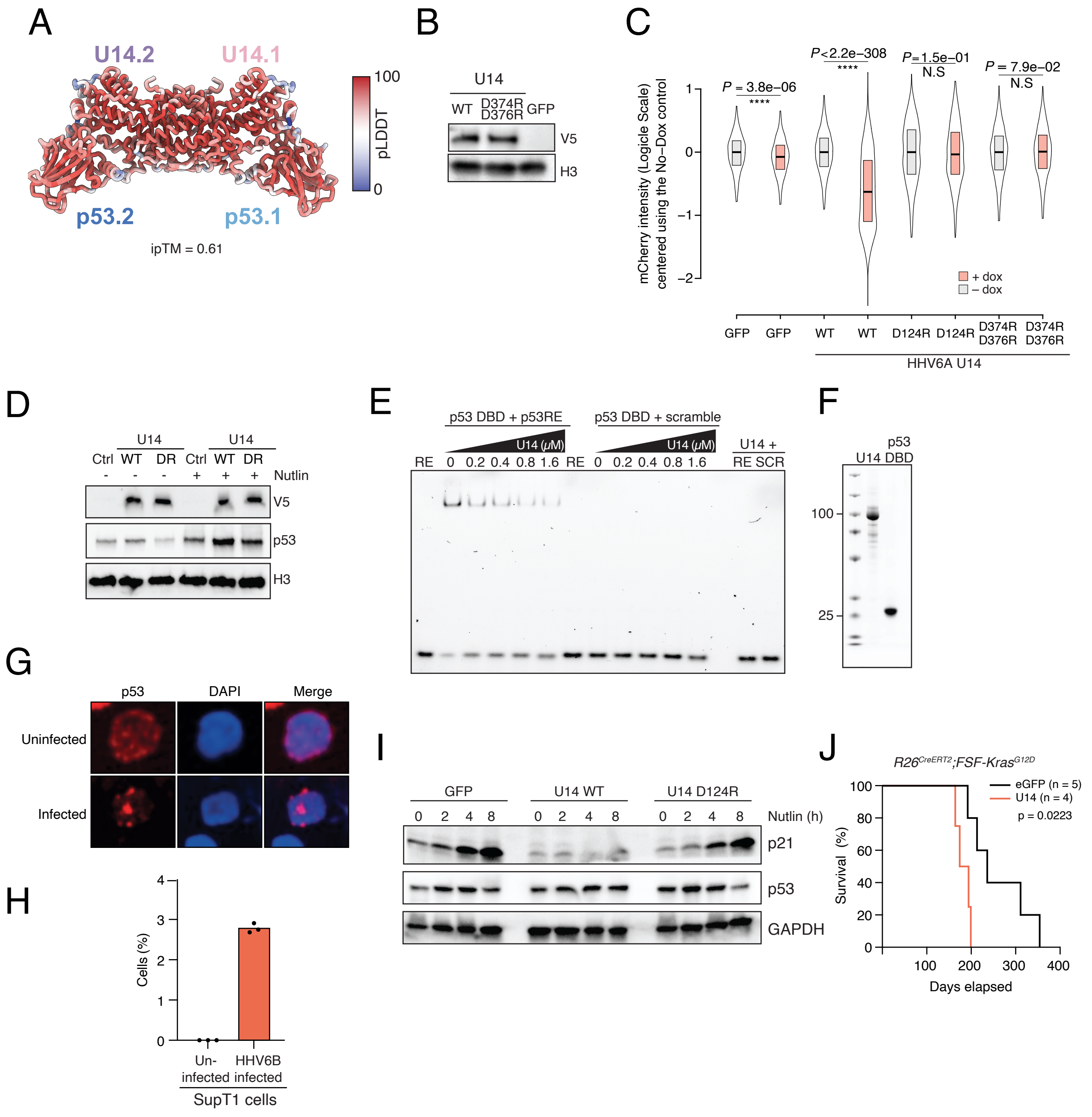
A
U14.2
U14.1
p53.2
p53.1
100
pLDDT
0
ipTM = 0.61
B
U14
WT
D374R D376R
GFP
V5
H3
C
mCherry intensity (Logicle Scale) centered using the No−Dox control
P = 3.8e−06
****
P<2.2e−308
****
P=1.5e−01
N.S
P = 7.9e−02
N.S
+ dox
− dox
GFP
GFP
WT
WT
D124R
D124R
D374R D376R
D374R D376R
HHV6A U14
D
U14
U14
Ctrl
WT
DR
Ctrl
WT
DR
Nutlin
V5
p53
H3
E
p53 DBD + p53RE
p53 DBD + scramble
U14 (μM)
U14 (μM)
U14 +
RE
0
0.2
0.4
0.8
1.6
RE
0
0.2
0.4
0.8
1.6
RE SCR
F
U14
p53 DBD
100
25
G
p53
DAPI
Merge
Uninfected
Infected
H
Cells (%)
Un-infected
HHV6B infected
SupT1 cells
I
GFP
U14 WT
U14 D124R
Nutlin (h)
p21
p53
GAPDH
J
R26CreERT2;FSF-KrasG12D
eGFP (n = 5)
U14 (n = 4)
p = 0.0223
Survival (%)
Days elapsed

Supplementary Figure 4. Validation of U14 from HHV6A as a p53 antagonist, related to Figure 4. (A) Predicted local distance difference test (pLDDT) scores for the U14–p53 complex structural model generated by AlphaFold 3. Interface predicted template modeling (ipTM) score for the structural model is indicated. (B) Western blot analysis of cells expressing V5-tagged U14, V5-tagged U14 D374R,D376R mutant, or an mCherry control. Lysates were immunoblotted with an anti-V5 antibody to confirm that the mutant protein is stably expressed. H3 served as a loading control. (C) Boxplots showing representative examples of the single-cell mCherry fluorescence distribution, complementing the median-based analysis presented in Figure 4. ORF expression was induced with dox (orange) or left untreated (grey) and where specified, cells were treated with 2.5 μM nutlin for 16 hours to stimulate p53 activity. The panels show results for wild-type U14 with its inactive mutants (D124R or D374R,D376R). The y-axis represents the Logicle-transformed mCherry fluorescence, centred to the median of the corresponding no-dox control population. Boxplots display the median and interquartile range. GFP serves as the negative control. Statistical significance was determined by comparing dox-treated versus untreated samples for each ORF. (D) Western blot analysis of cells expressing GFP, V5-tagged U14, or V5-tagged U14 D124R mutant, treated with or without 2.5 μM nutlin for 1 hour. Blots were probed with antibodies against V5, p53, and Histone H3 as a loading control. (E) Electrophoretic mobility shift assay (EMSA) showing the inhibition of p53 DNA-binding by U14. Purified p53 DBD (400 nM) was incubated with a FAM-labelled p53 response element (p53RE or RE) or a scrambled control probe (5 nM) in the presence of increasing concentrations of purified U14 (200, 400, 800, and 1600 nM). Control reactions included probe alone, p53 alone, U14 alone (400 nM) with either p53RE or scrambled probes. Samples were resolved on native 6% polyacrylamide gels and DNA was detected by fluorescence imaging. (F) Coomassie blue-stained SDS-PAGE gel showing purified U14 and p53 DBD proteins used for EMSA. (G) Uninfected or HHV6B-infected SupT1 cells were stained with anti-p53 (red) and DAPI (blue). (H) Quantitation of cells positive for nuclear p53 foci in uninfected and HHV6B-infected SupT1 cells. (I) Western blot analysis of mouse LLC1 cells expressing GFP, U14, or the U14 D124R mutant, treated with nutlin for the indicated time points (0–8 hours). Blots were probed for p21 and p53, with GAPDH serving as a loading control. (J) Survival curve of Rosa-CreERT2; FSF-KrasG12D mice following inhalation of lentivirus co-expressing U14 (orange, n = 4) or eGFP control (black, n = 5). Days elapsed reflects time post-tamoxifen induction. *padj < 0.05, **padj < 0.01, ***padj < 0.001, **** padj < 0.0001.

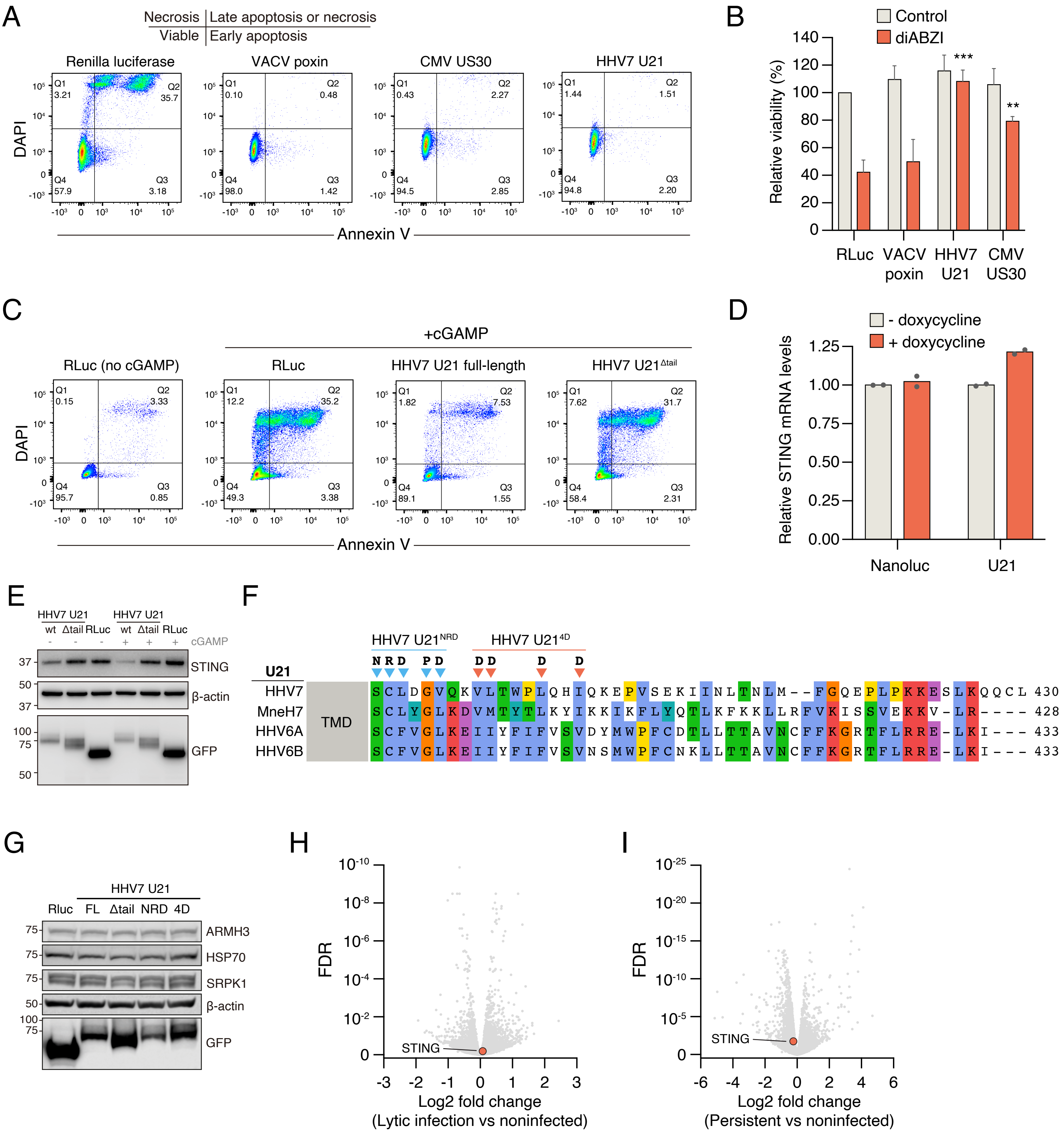
A
Necrosis
Late apoptosis or necrosis
Viable
Early apoptosis
Renilla luciferase
VACV poxin
CMV US30
HHV7 U21
DAPI
Annexin V
B
Control
diABZI
Relative viability (%)
RLuc
VACV poxin
HHV7 U21
CMV US30
C
+cGAMP
RLuc (no cGAMP)
RLuc
HHV7 U21 full-length
HHV7 U21Δtail
D
- doxycycline
+ doxycycline
Relative STING mRNA levels
Nanoluc
U21
E
HHV7 U21
wt
Δtail
RLuc
cGAMP
STING
β-actin
GFP
F
HHV7 U21NRD
HHV7 U214D
U21
HHV7
MneH7
HHV6A
HHV6B
TMD
G
HHV7 U21
Rluc
FL
Δtail
NRD
4D
ARMH3
HSP70
SRPK1
β-actin
GFP
H
FDR
STING
Log2 fold change
(Lytic infection vs noninfected)
I
FDR
STING
Log2 fold change
(Persistent vs noninfected)

Supplementary Figure 5. Validation of U21 as a potent inhibitor of STING signaling, related to Figure 5. **(**A) U937 cells stably expressing dox-inducible GFP-tagged Renilla luciferase, VACV poxin, HCMV US30, or HHV7 U21 were induced with dox followed by 2',3'-cGAMP treatment and analyzed for apoptosis with Annexin V and DAPI staining. (B) The same constructs were analyzed for viability CellTiter-Glo after treating the cells with the non-nucleotide STING agonist diABZI. (One-way ANOVA with Dunnett's comparisons test ***padj < 0.001, **padj < 0.01, n = 3). (C) Cells expressing full-length HHV7 U21 or U21Δtail were analyzed for apoptosis after cGAMP treatment as in (A). (D) STING mRNA levels were assessed by qRT-PCR in cells expressing Nanoluc-GFP or U21-GFP. (E) STING protein levels were analyzed by western blotting in cells expressing the indicated GFP-tagged constructs after treating the cells with cGAMP or vehicle. (F) Alignment of the C-terminal cytoplasmic tails of U21 from HHV7, HHV6A, HHV6B and *Macaca nemestrina* herpesvirus 7 (MneH7). (G) Western blot analysis of ARMH3, Hsp70 and SRPK1 protein levels in cells expressing U21 or indicated mutants. Note that the β-actin and GFP blots are the same as in Figure 5I as these proteins were analyzed in the same gel run. (H) RNAseq of SupT1 cells freshly infected with HHV7 compared to uninfected SupT1 cells. (I) RNAseq of SupT1 cells persistently infected with HHV7 compared to uninfected SupT1 cells. STING is highlighted in orange.

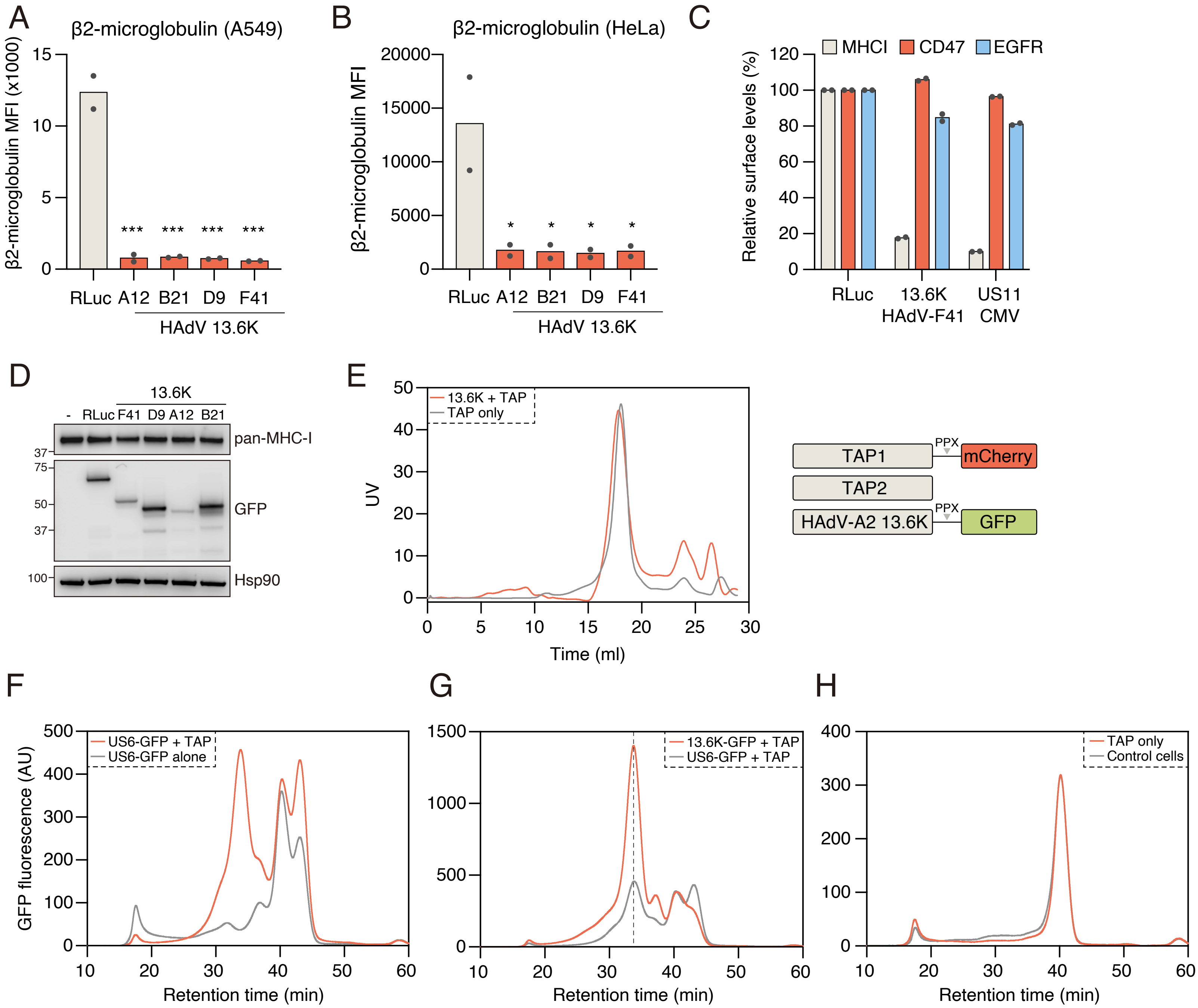

A
β2-microglobulin (A549)
β2-microglobulin MFI (x1000)
***
RLuc
A12
B21
D9
F41
HAdV 13.6K
B
β2-microglobulin (HeLa)
β2-microglobulin MFI
*
RLuc
A12
B21
D9
F41
HAdV 13.6K
C
MHCI
CD47
EGFR
Relative surface levels (%)
RLuc
13.6K HAdV-F41
US11 CMV
D
13.6K
-
RLuc
F41
D9
A12
B21
pan-MHC-I
GFP
Hsp90
E
13.6K + TAP
TAP only
UV
Time (ml)
TAP1
PPX
mCherry
TAP2
HAdV-A2 13.6K
PPX
GFP
F
US6-GFP + TAP
US6-GFP alone
GFP fluorescence (AU)
Retention time (min)
G
13.6K-GFP + TAP
US6-GFP + TAP
Retention time (min)
H
TAP only
Control cells
Retention time (min)

Supplementary Figure 6. Validation of 13.6K proteins as novel TAP inhibitors, related to Figure 6. (A-B) A549 cells (A) and HeLa cells (B) expressing indicated GFP-tagged constructs were assessed for cell surface β2-microglobulin levels by flow cytometry. (One-way ANOVA with Dunnett's comparisons test ***padj < 0.001, *padj < 0.05). (C) HeLa cells expressing GFP-tagged HAdV-F41 13.6K or HCMV US11, a known inhibitor of MHC-I surface display, were analyzed for cell surface MHC-I, EGFR, or CD47 levels by flow cytometry. (D) Western blot analysis of total MHC-I levels in HeLa cells expressing indicated 13.6K-GFP constructs. (E) TAP1-mCherry and TAP2 with or without 13.6K-GFP were expressed in HEK293S cells and purified with anti-mCherry (TAP1 and TAP2 only) or anti-GFP (TAP1/TAP2/13.6K) affinity chromatography and size exclusion chromatography. The plot shows the SEC elution profile of both purifications. PPX, PreScission protease cleavage site. (F) HCMV US6-GFP was transfected into HEK293S TAP1 knockout cells with or without untagged TAP1 and TAP2 followed by FSEC. (G) Comparison of 13.6K-GFP and US6-GFP FSEC elution profiles. (H) FSEC profiles of untransfected cells and cells transfected with untagged TAP1 and TAP2. The peak at 40 minutes corresponds to nonspecific fluorescence present in HEK293S cells.
